# Highly Efficient Transpeptidase-Catalyzed Isopeptide Ligation

**DOI:** 10.1021/jacs.4c11964

**Published:** 2024-12-23

**Authors:** Simon J. de Veer, David J. Craik, Fabian B. H. Rehm

**Affiliations:** † Institute for Molecular Bioscience, Australian Research Council Centre of Excellence for Innovations in Peptide and Protein Science, The University of Queensland, Brisbane, Queensland 4072, Australia; ‡ Medical Research Council Laboratory of Molecular Biology, Cambridge CB2 0QH, UK

## Abstract

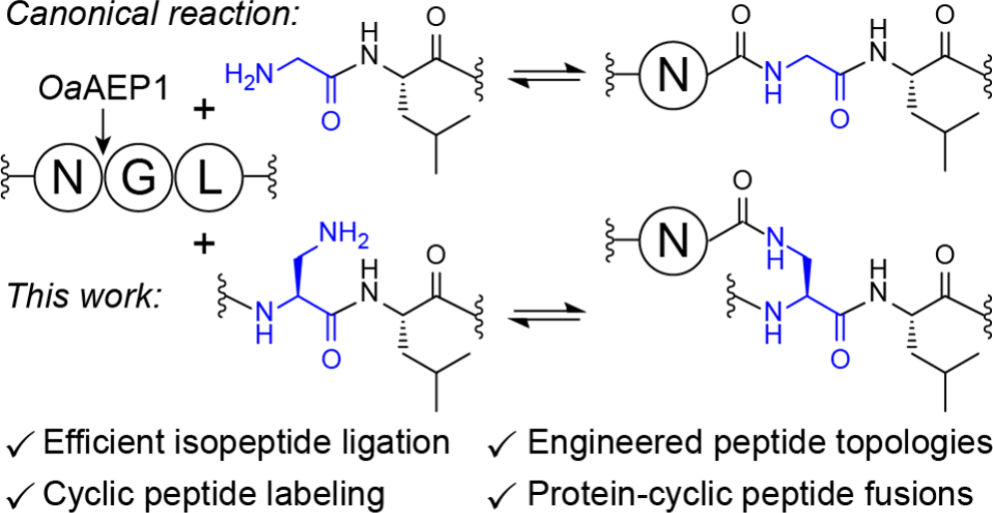

Transpeptidases are specialized enzymes that have evolved for site-selective modification of peptides and proteins at their backbone termini. Approaches for adapting transpeptidases to catalyze side chain modifications are substantially more restricted, and typically rely on large recognition tags or require specific reaction conditions that are not easily compatible with broader applications. Here we show that the engineered asparaginyl ligase *Oa*AEP1 catalyzes direct isopeptide ligation by accepting an internal 2,3-diaminopropionic acid (Dap) residue adjacent to Leu, a motif that mimics the canonical N-terminal Gly-Leu substrate. These reactions proceed efficiently at near-neutral pH without any required additives, enabling straightforward formation of diverse isopeptide-linked products under simple reaction conditions. We demonstrate that *Oa*AEP1-catalyzed isopeptide ligation can be utilized for site-selective side chain labeling at an introduced Dap residue with minimal off-target labeling of Lys residues. Additionally, we generate engineered peptide topologies via intramolecular side chain-to-tail cross-links and produce direct protein–cyclic peptide fusions via efficient intermolecular ligation. We also show that *Oa*AEP1-catalyzed isopeptide ligation extends to d-peptide acceptors containing a retro-inverso d-Leu-d-Dap motif. This capability further expands the range and complexity of isopeptide-linked products that can be accessed with *Oa*AEP1, which we exemplify by forming a hybrid d-/l- bicyclic peptide topology where both termini are linked to internal side chains.

## Introduction

Transpeptidases have emerged as valuable tools for peptide and protein modification due to their broad versatility and exceptional site-selectivity, which surpass equivalent chemical approaches.^1^ These enzymes, including sortases, asparaginyl ligases and subtiligases,^2−4^ act by cleaving substrates at a precise recognition sequence then ligating the retained substrate fragment to the N-terminal primary amine of an acceptor sequence to form a peptide bond. Such a reaction is ideally suited to modifying peptide and protein termini, and a wide range of approaches has been established for using transpeptidases to label protein N- or C-termini, generate head-to-tail cyclic peptides or proteins, or ligate two separate proteins at their termini to form defined chimeric constructs.^1−4^ Although reactions that are largely restricted to substrate N- and C-termini have the benefit of specificity, this trait equally represents a fundamental limitation regarding the types of modifications that can be directly accessed.

Enzymatic approaches for side chain modification of peptides and proteins typically employ dedicated ligases or transferases, such as biotin ligase,^5^ transglutaminases^6^ or ubiquitin-conjugating enzymes.^7^ However, these enzymes have several drawbacks for broader application, including large and bulky recognition motifs, dependence on cofactors, limited substrate scope or low efficiency for noncanonical reactions. An alternative nonenzymatic strategy involves split bacterial adhesins, such as the SpyTag/SpyCatcher system,^8^ which enables rapid and spontaneous isopeptide ligation between specific Lys and Asp side chains, but the reconstituted adhesin domain (≥97 residues) is ultimately retained in the product.

Until recently, approaches for transpeptidase-based side chain modification were substantially more restricted. An initial approach was inspired by the natural ability of sortase homologues to cross-link proteins during bacterial pili assembly. This reaction involves a Lys-containing pilin domain that was adapted as a tag for site-selective isopeptide ligation using sortase,^9^ but the length of this motif (≥17-amino acids) restricts where it can be practically introduced–thus far this approach appears to be restricted to protein substrates with pilin domains introduced in accessible terminal regions. This limitation has been circumvented by the use of genetic code expansion to incorporate synthetic Lys derivatives bearing a side chain dipeptide to provide a second N-terminus available for standard transpeptidation reactions.^10,11^ However, adapting transpeptidases for direct isopeptide ligation that leaves a minimal footprint in the product remains a major outstanding challenge.

Recently, the C247A mutant of the asparaginyl ligase *Oa*AEP1^12^ (hereafter referred to as *Oa*AEP1) was shown to catalyze isopeptide ligation by accepting an internal Lys residue when adjacent to a Leu residue.^13^ This new capability draws on the promiscuity of *Oa*AEP1 for amine nucleophiles, which extends beyond the canonical N-terminal Gly-Leu motif^14^ and includes primary amine-containing small molecules,^15^ N-terminal secondary amines,^16^ and C-terminal mimetics of the N-terminal Gly-Leu motif.^17^ To ligate these nucleophiles to an acyl donor substrate, *Oa*AEP1 first cleaves the substrate at an Asn↓Gly-Leu recognition sequence to form a thioester-linked acyl-enzyme intermediate in which the (Xaa)_*n*_-Asn fragment is retained. In conventional transpeptidation reactions, this intermediate is resolved via nucleophilic attack by the N-terminal primary amine of a Gly-Leu-(Yaa)_*n*_ sequence to form a C-to-N ligated product. Other nucleophiles can be incorporated via a similar reaction, with the acyl-enzyme intermediate resolved via nucleophilic attack by the alternative amine-containing nucleophile.

Among the amine nucleophiles accepted by *Oa*AEP1, the Lys ε-amine is one of the least optimal and, to reach reasonable levels of product, the reaction requires very specific conditions to suppress two competing nucleophiles.^13^ First, the byproduct released upon cleavage of the acyl donor substrate must be quenched (e.g., by supplementing the reactions with Ni^2+^ to quench a GLH byproduct)^18^ to avoid preferential incorporation of the cleaved byproduct, which would lead to unwanted production of the starting material, instead of the target Lys-Leu acceptor to yield the desired product. Second, intermolecular ligations must be carried out at pH ≥ 8 to suppress the competing hydrolysis reaction, which is exacerbated by quenching the GLH byproduct, thereby necessitating much higher concentrations of enzyme as this pH is suboptimal for *Oa*AEP1. These requirements restrict practical application of *Oa*AEP1-catalyzed Lys-targeted isopeptide ligation in certain cases.

We anticipated that increasing the reactivity of the Lys-Leu acceptor would substantially improve the efficiency of asparaginyl ligase-catalyzed isopeptide ligation and potentially enable new types of modifications that were otherwise challenging with Lys. We also envisaged that utilizing an acceptor sequence that does not occur within the natural proteome might enable exquisite site-selectivity, irrespective of the peptide or protein sequence (i.e., even in the presence of endogenous Lys residues, particularly Lys adjacent to Leu). These considerations led us to explore Lys analogs–we were particularly interested in 2,3-diaminoproponic acid (Dap) as the side chain β-amine of this analog has a substantially lower p*K*_a_ than the Lys ε-amine,^19^ making it sufficiently nucleophilic to replace the catalytic Ser or Cys in diverse hydrolytic enzymes and enable capture of stable, amide-linked acyl-enzyme complexes.^20,21^

## Results and Discussion

To characterize the scope of amine nucleophiles accepted by *Oa*AEP1 for isopeptide ligation, we first compared product formation for Lys and nonproteinogenic Lys analogs that alter the position of the side chain amine relative to the peptide backbone and the adjacent Leu residue. We synthesized Nα-acetylated peptides (sequence Ac-GXLGV) where *X* = Lys, ornithine (Orn), 2,4-diaminobutyric acid (Dab), or Dap, and tested their ligation to a model acyl donor substrate (Ac-GWRNGLH, 100 μM) using 5 equiv. Ac-GXLGV, as described previously.^13^ However, to assess isopeptide ligation under less stringent reaction conditions than the prior study, we did not use a quenching approach (i.e., Ni^2+^ was not added to the reactions) and the pH was lowered from 8.5 to 7.5, which also allowed the enzyme concentration to be decreased from 1 μM to 200 nM.

**Figure 1:**
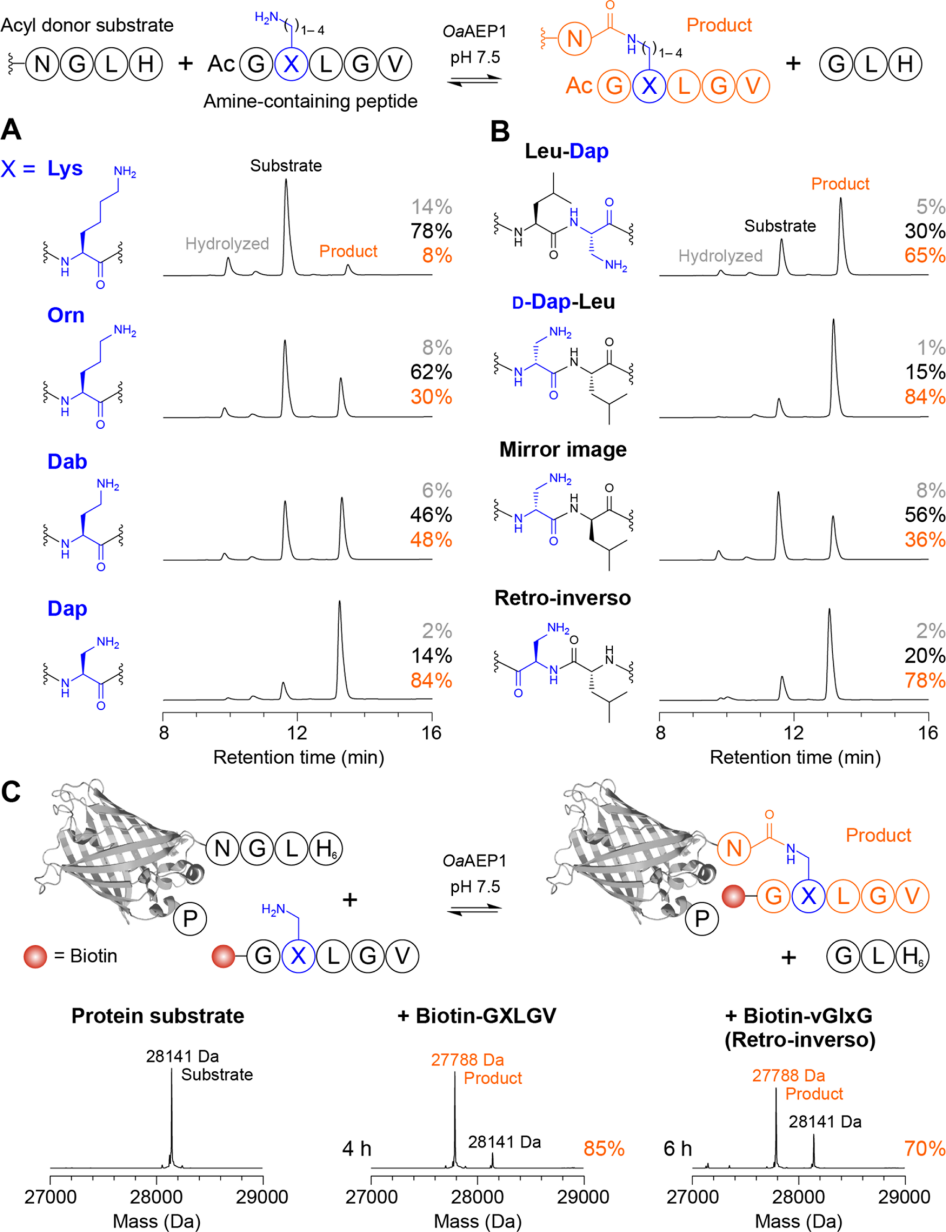
Efficient asparaginyl ligase-catalyzed isopeptide ligation at near-neutral pH. (A) Nα-acetylated peptides bearing a side chain amine (Ac-GXLGV where *X* = Lys, ornithine (Orn), 2,4-diaminobutyric acid (Dab) or 2,3-diaminopropionic acid (Dap), 500 μM, 5 equiv) were ligated to an NGL-containing substrate (Ac-GWRNGLH, 100 μM) using 200 nM *Oa*AEP1 in 100 mM 4-(2-hydroxyethyl)-1-piperazineethanesulfonic acid (HEPES) pH 7.5 (45 min at 25 °C). Shown are analytical reverse-phase high-performance liquid chromatography (RP-HPLC) traces (A280 nm). Peaks for acyl donor substrate (black), product (orange), and hydrolyzed substrate (gray) are indicated, as well as the percentage of each species. Spectra from matrix-assisted laser desorption ionization time-of-flight mass spectrometry (MALDI-TOF MS) are shown in Figure S1A. (B) Isopeptide ligation with modified Dap substrates. Reactions were carried out as in (A) using Ac-GL[Dap]GV (Leu-Dap), Ac-G[d-Dap]LGV (d-Dap-Leu), Ac-G[d-Dap]lGv (mirror image) or Ac-vGl[d-Dap]G (retro-inverso). Spectra from MALDI-TOF MS are shown in Figures S1B. (C) Isopeptide ligation at the C-terminus of a protein. eGFP with a C-terminal NGL-His_6_ extension was labeled with biotin-G[Dap]LGV or biotin-vGl[d-Dap]G in reactions comprising 25 μM protein, 250 μM peptide (10 equiv) and 500 nM *Oa*AEP1 in 100 mM HEPES pH 7.5 (reaction times as indicated). Shown are reconstructed spectra from electrospray ionization mass spectrometry (ESI-MS) with the observed substrate (black) and product (orange) masses indicated. Additional time points are shown in Figure S12.

As anticipated, at near-neutral pH and without GL byproduct quenching, only low levels of product (8%) were observed for the Lys-containing peptide Ac-GKLGV (Figures 1A and S1A). However, positioning the side chain amine closer to the peptide backbone led to progressive improvements in yield of the isopeptide-linked product (Figure 1A). The highest conversion to product was observed for the Dap-containing peptide (84% conversion within 45 min), which reached a similar yield to a conventional transpeptidation reaction using a GLRL nucleophile under the same conditions (83%, Figure S2). This exceeds the yield previously reported for Ac-GKLGV (76%) under more stringent conditions (Ni^2+^ quenching, pH 8.5, 1 μM enzyme, 90 min).^13^ When ligation of the Ac-GXLGV peptides was examined in the presence of Ni^2+^, we observed higher levels of product formation (Figures S3 and S4), including quantitative conversion for Ac-G[Dap]LGV, but hydrolysis of the acyl donor substrate was clearly yield-limiting for the Lys-containing acceptor at near-neutral pH. For Ac-G[Dap]LGV, we also found that quenching the GL byproduct facilitated efficient isopeptide ligation at lower concentrations of acceptor (Figures S5 and S6), such that just 1.5 equiv of Ac-G[Dap]LGV was sufficient to reach >90% conversion to product.

We next examined further modification of the Xaa-Leu sequence within the acceptor peptide. Initially, we tested placing Leu prior to the Xaa residue bearing a side chain amine, as Leu-Lys was also shown to be compatible with *Oa*AEP1-catalyzed isopeptide ligation.^13^ Without GL byproduct quenching, product formation was also observed for the Ac-GLXGV peptides (Figures S3 and S4), but generally with lower yields than the corresponding Ac-GXLGV peptides. Interestingly, formation of the isopeptide-linked product at Leu-Dap was more efficient than for Dab, Orn, or Lys in the more favored Xaa-Leu configuration (Figures S3 and S4), further highlighting that Dap was preferred in these reactions.

To test the importance of the adjacent Leu residue, we synthesized a series of Dap-containing peptides (sequence: Ac-AG[Dap]XGA) where the residue following Dap was varied to Ile, Phe, Gly, Pro, Gln, or Ser. These residues were selected based on their diverse biophysical properties and the previously reported specificity data for Lys-Xaa.^13^ We found that hydrophobic residues Ile and Phe were also compatible with efficient isopeptide ligation at Dap-Xaa (Figures S7 and S8), which parallels the specificity of *Oa*AEP1 for isopeptide ligation at Lys-Xaa and transpeptidation at the canonical N-terminal Gly-Xaa motif. These findings indicate that internal Dap-Leu represents a genuine substrate mimetic. We also examined specificity effects for the residue preceding Dap using peptides based on the sequence Ac-AX[Dap]LGA. Replacing Gly in the previous substrates with Ile, Phe, Pro, Gln, or Ser did not affect the product yield (Figures S7 and S9), suggesting that Dap-Leu can be introduced in diverse sequence contexts.

We subsequently characterized *Oa*AEP1-catalyzed isopeptide ligation using acceptor peptides with altered stereochemistry, focusing on Dap-containing peptides due to their higher conversion to product. We found that inverting the stereochemistry of the Dap residue (l- to d-) in Ac-GXLGV did not alter conversion to product (Figure 1B). Encouraged by this result, we examined ligation of a mirror image acceptor peptide (Ac-Gly-dap-leu-Gly-val; d-amino acids are indicated by lower case, three-letter code) and found that product could be formed (36% conversion, Figure 1B), which was improved upon quenching the competing GL byproduct (80% conversion, Figures S10 and S11). Based on our earlier finding that internal Dap-Leu is a genuine mimetic for N-terminal Gly-Leu (Figures S7–S9), we hypothesized that a retro-inverso acceptor containing a d-Leu-d-Dap motif might enable improved ligation of d-peptides as the conformation of key side chain determinants would be maintained. Reactions with the retro-inverso acceptor (Ac-val-Gly-leu-dap-Gly) generated high levels of isopeptide-linked product (78% conversion, without GL quenching) that were comparable to the optimal (all-l) acceptor (84% conversion, Figure 1A,B), thereby supporting our hypothesis.

Having shown that Dap adjacent to a Leu residue enables efficient peptide-based isopeptide ligation under mild conditions (near-neutral pH, without GL quenching), we tested whether the reaction could be applied to protein labeling. We synthesized a biotin-G[Dap]LGV peptide substrate and examined labeling of a model protein, enhanced green fluorescent protein (eGFP), with a C-terminal NGL-H_6_ extension. ESI-MS analysis revealed that the isopeptide-linked labeled protein was readily formed, reaching 85% conversion to product within 4 h (Figures 1C and S12). We subsequently examined whether an all-d peptide could be enzymatically conjugated to a protein via an isopeptide bond. For these reactions, we used a biotinylated retro-inverso peptide (biotin-val-Gly-leu-dap-Gly) and found that the isopeptide-linked eGFP-d-peptide fusion product was also formed (Figure 1C), albeit with lower efficiency (70% conversion after 6 h) than the acceptor peptide comprised of l-amino acids.

**Figure 2:**
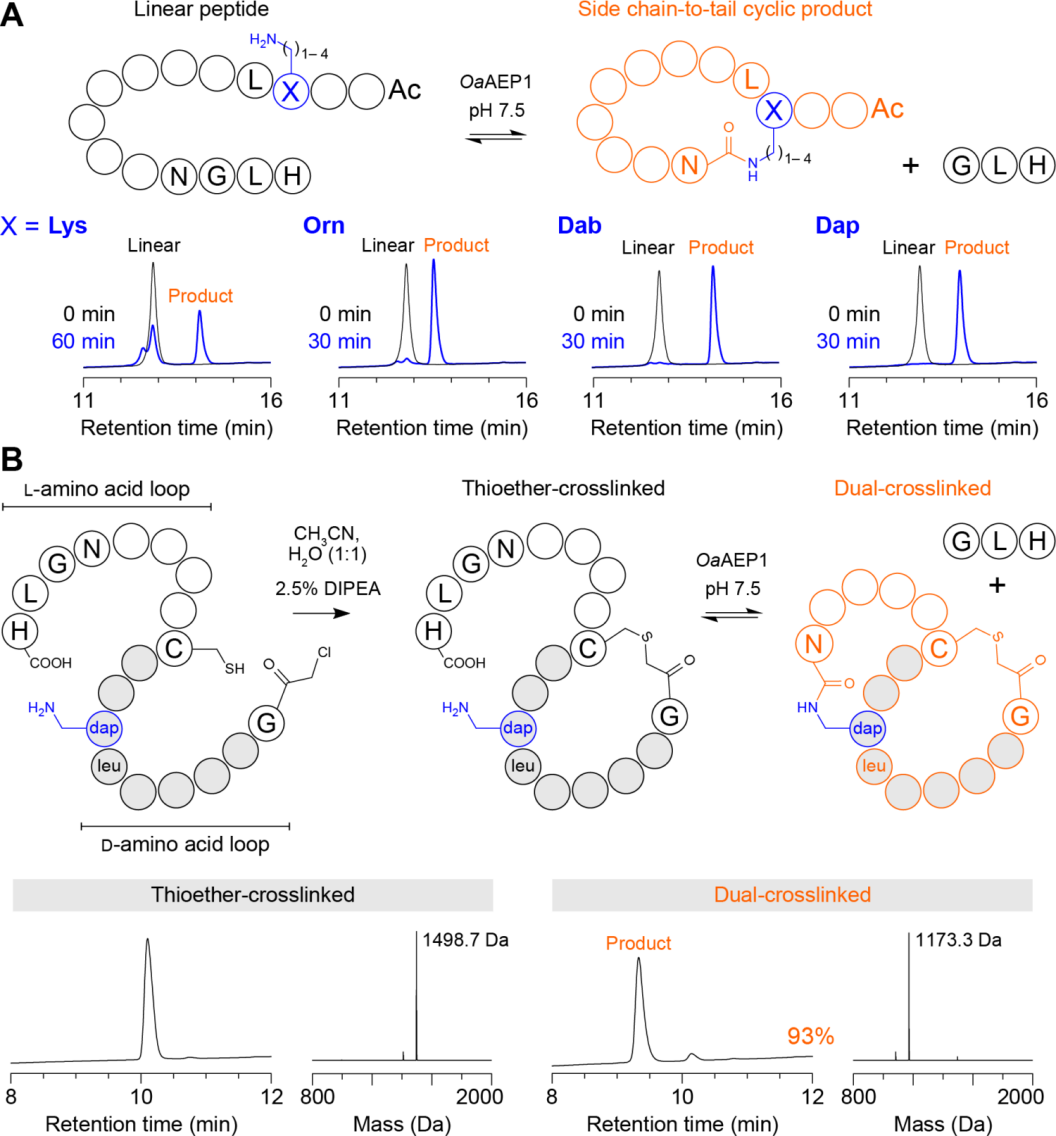
Engineered peptide topologies via intramolecular isopeptide ligation. (A) Isopeptide-linked macrocycles with cross-links of varying lengths. Model peptides based on a linear template (sequence: Ac-YGXLTVpGLTRNGLH, *p* = d-Pro and *X* = Lys, Orn, Dab or Dap) were cyclized in reactions comprising 50 μM peptide and 100 nM *Oa*AEP1 in 100 mM HEPES pH 7.5 (30 or 60 min at 25 °C). Shown is an overlay of analytical RP-HPLC traces (A280 nm) of the starting material (black) and reaction products (blue). Peaks for the linear peptide substrate and side chain-to-tail cyclic product are indicated. Spectra from MALDI-TOF MS are shown in Figure S13. (B) *Oa*AEP1-catalyzed formation of a hybrid d-/l- bicyclic peptide topology. A linear peptide with an N-terminal d-amino acid segment (containing a d-Leu-d-Dap motif) and a C-terminal l-amino acid segment (containing an NGL recognition motif) was first head-to-side chain cyclized via a thioether cross-link between the terminal chloroacetyl-Gly residue and an internal Cys residue (sequence: c[GGasGl[d-Dap]GasGGC]GNGLH). The purified thioether-cross-linked peptide served as a substrate for *Oa*AEP1-catalyzed isopeptide ligation in reactions comprising 50 μM peptide and 200 nM *Oa*AEP1 in 100 mM HEPES pH 7.5 (60 min at 25 °C) to form the dual-cross-linked peptide containing an N-terminal d-amino acid loop and a C-terminal l-amino acid loop. Shown are analytical RP-HPLC traces (A214 nm) and reconstructed spectra from ESI-MS of the purified thioether-cross-linked substrate (left) and the crude reaction products from *Oa*AEP1-catalyzed isopeptide ligation to generate the dual-cross-linked product (right).

Another application of *Oa*AEP1-catalyzed isopeptide ligation involves intramolecular reactions for generating engineered peptide topologies. Given that Orn, Dab, and Dap were compatible with intermolecular peptide ligation, we examined whether these residues could also facilitate intramolecular side chain-to-tail cyclization and, thus, generate isopeptide-linked macrocycles with cross-links of varying lengths. We synthesized Nα-acetylated model peptides containing an internal Xaa-Leu sequence and a C-terminal NGL recognition motif (sequence: Ac-YGXLTVpGLTRNGLH, where *p* = d-Pro and *X* = Lys, Orn, Dab or Dap) based on a previously reported β-hairpin peptide^22^ and assessed cyclization using 50 μM substrate and 100 nM *Oa*AEP1 in 100 mM HEPES pH 7.5 (25 °C for 30 or 60 min, as indicated in Figure 2A). Although cyclization of the Lys-containing peptide was less efficient than for Orn, Dab, or Dap, we found that each side chain-to-tail cyclic product could be formed (Figures 2A and S13), thus demonstrating enzymatic formation of isopeptide-linked macrocycles with cross-links of three (Dap) to six (Lys) atoms between the respective α-carbons.

The structure of the cyclic product generated by isopeptide ligation for the Dap β-hairpin peptide was verified by ^1^H two-dimensional (2D) nuclear magnetic resonance (NMR) spectroscopy. New nuclear Overhauser effect cross-peaks were observed between the Dap3 H^γ^ proton and the Asn12 H^α^ and H^N^ protons, consistent with side chain-to-tail cyclization via an isopeptide bond (Figure S14). Additionally, we examined varying the concentration of *Oa*AEP1 for cyclization of the Dap β-hairpin peptide (50 μM) and found that 25 nM (0.0005 equiv) was sufficient to maintain near-quantitative conversion to product within 60 min (Figure S15). We also tested side chain-to-tail cyclization of a substrate with an entirely different topology, which we exemplified using a stapled helical peptide. This substrate was produced with an N-terminal Dap-Leu motif and a C-terminal NGL recognition motif (sequence: Ac-YG[Dap]LGAREA[hCys]ARE[hCys]AAREGNGLH), and was stapled with 1,3-dichloroacetone via the two hCys residues.^23^ We verified the helical conformation of the substrate peptide by NMR spectroscopy and secondary Hα chemical shift measurements (−0.2 ppm for eight consecutive residues, Figure S16). In reactions comprising 50 μM peptide substrate and 200 nM *Oa*AEP1, we observed near-quantitative conversion to product within 60 min (Figure S16), although the additional constraint of side chain-to-tail cyclization did appear to affect peptide helicity as indicated by secondary Hα chemical shift measurements for the product (Figure S16).

We next explored formation of a more complex, bicyclic topology generated by cross-linking each peptide terminus to a separate internal side chain. We envisaged that such a topology could be formed in two reaction steps: (1) a chemical head-to-side chain ligation, followed by (2) an enzymatic side chain-to-tail ligation using *Oa*AEP1. To test this hypothesis, we synthesized a model peptide bearing an N-terminal chloroacetyl moiety and an internal Cys residue to enable formation of a thioether cross-link in the first reaction, as well as a C-terminal NGL sequence and an internal Dap-Leu motif for isopeptide ligation in the second reaction. The thioether-cross-linked intermediate was readily formed in CH_3_CN/H_2_O (1:1) containing 2.5% *N*,*N*-diisopropylethylamine (DIPEA), and then purified before carrying out the isopeptide ligation using 50 μM substrate and 200 nM *Oa*AEP1 in 100 mM HEPES pH 7.5 (60 min at 25 °C). This reaction resulted in near-quantitative conversion to the dual-cross-linked, bicyclic peptide (95% conversion to product, Figure S17).

To extend this concept further, we considered that the retro-inverso d-Leu-d-Dap motif is also efficiently accepted by *Oa*AEP1 for isopeptide ligation. This observation suggested that it might also be possible to enzymatically generate a hybrid d-/l- bicyclic topology, where one loop of the peptide is comprised of d-amino acids and the other is comprised of l-amino acids. To test this possibility, we synthesized an analog of the previous bicyclic peptide substrate whereby l-amino acids in the first loop were replaced with d-amino acids and the positions of Dap and Leu were reversed (sequence: ^ClAc^GGasGl[d-Dap]GasGGCGNGLH). After forming the thioether-cross-linked intermediate, the purified peptide was incubated with *Oa*AEP1 (conditions as above) to efficiently generate the dual-cross-linked hybrid d-/l- bicyclic topology (93% conversion, Figure 2B).

**Figure 3:**
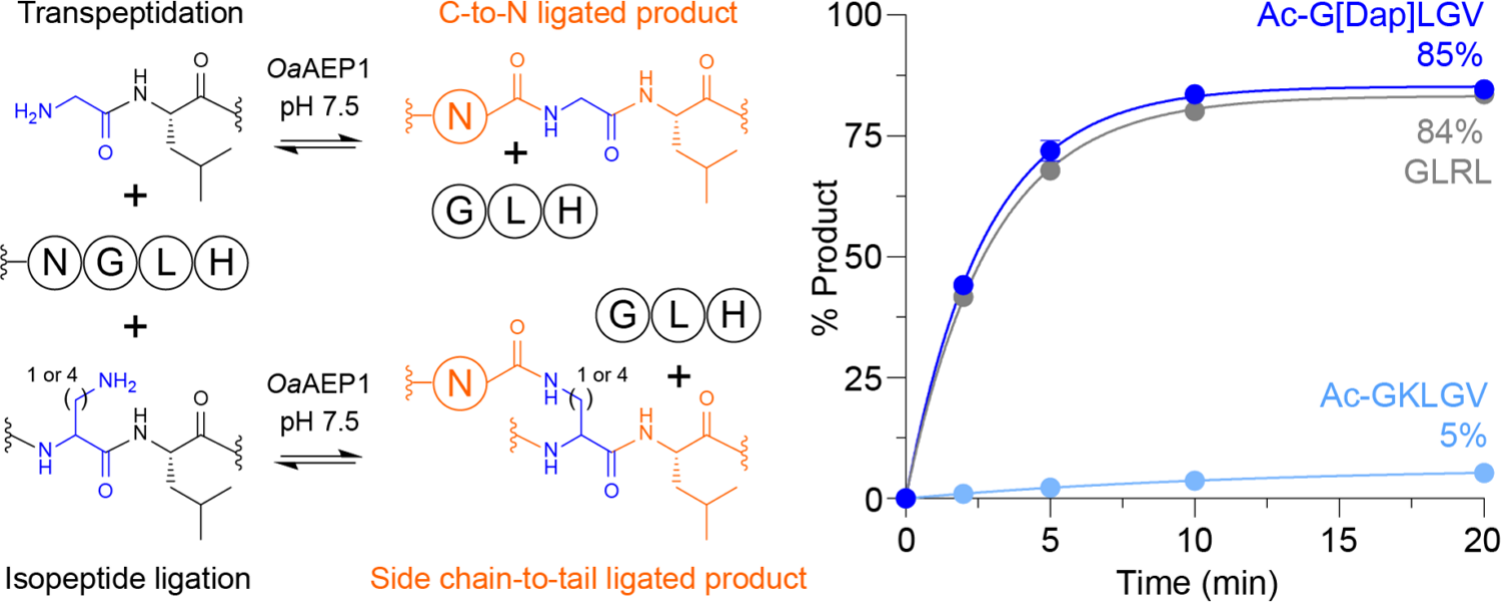
Time course for *Oa*AEP1-catalyzed isopeptide ligation at Dap-Leu compared to a conventional transpeptidation reaction at N-terminal GL. Reactions comprised 100 μM acyl donor substrate (Ac-GWRNGLH), 500 μM peptide nucleophile (Ac-G[Dap]LGV, Ac-GKLGV or GLRL, 5 equiv) and 200 nM *Oa*AEP1 in 100 mM HEPES pH 7.5 (25 °C) and were analyzed by RP-HPLC (Figure S18). Data points represent the mean ± standard deviation (*n* = 3, error bars are within the size of the data point symbols) for Ac-G[Dap]LGV (dark blue), Ac-GKLGV (light blue) or GLRL (gray) and were fit to one-phase association curves in GraphPad Prism 10.

Based on our observations that Dap-containing peptides were highly efficient acceptors for isopeptide ligation in different substrate contexts, we were interested in comparing product formation over time for this noncanonical reaction relative to a conventional transpeptidation reaction with a GL nucleophile. To this end, we monitored ligation of Ac-G[Dap]LGV to a model acyl donor substrate (Ac-GWRNGLH) compared to GLRL and the previously reported Ac-GKLGV acceptor (Figure S18). Figure 3 shows that, with model peptides, isopeptide ligation at internal Dap-Leu proceeds with near-identical kinetics to a transpeptidation reaction at N-terminal GL under equivalent reaction conditions. By contrast, only low levels of product were observed for Ac-GKLGV. As these reactions did not employ a quenching approach, the time course data also highlight that Dap-Leu is a substantially better competitor than Lys-Leu with respect to the GL byproduct released upon cleavage of the acyl donor substrate. This finding is consistent with our earlier observations that isopeptide ligation at Dap-Leu is substantially less dependent on GL byproduct quenching than reactions at Lys-Leu (Figures 1A and S1A).

To further characterize the kinetic parameters for isopeptide ligation using Dap-containing acceptors, we analyzed Michaelis–Menten kinetics for the reaction between Ac-G[Dap]LGV and the model acyl donor substrate Ac-GWRNGLH (Figure S19). Under mild reaction conditions (pH 7.5, no additives), we determined a *K*_M_ of 167 μM and a catalytic efficiency (*k*_cat_/*K*_M_) of 3.6 × 10^4^ M^–1^ s^–1^, which represents more than an order of magnitude improvement in *k*_cat_/*K*_M_ compared to isopeptide ligation at Lys-Leu (where Ni^2+^ is required to quench the GL byproduct).^13^ Given that quenching the GL byproduct enabled efficient isopeptide ligation at lower concentrations of Ac-G[Dap]LGV (Figures S5 and S6), we also determined kinetic constants in the presence of Ni^2+^ for comparison (Figure S19). We found that quenching the GL byproduct improved *k*_cat_/*K*_M_ for Ac-G[Dap]LGV further (7.6 × 10^4^ M^–1^ s^–1^) by lowering *K*_M_ (76 μM), whereas *k*_cat_ remained essentially unchanged.

**Figure 4:**
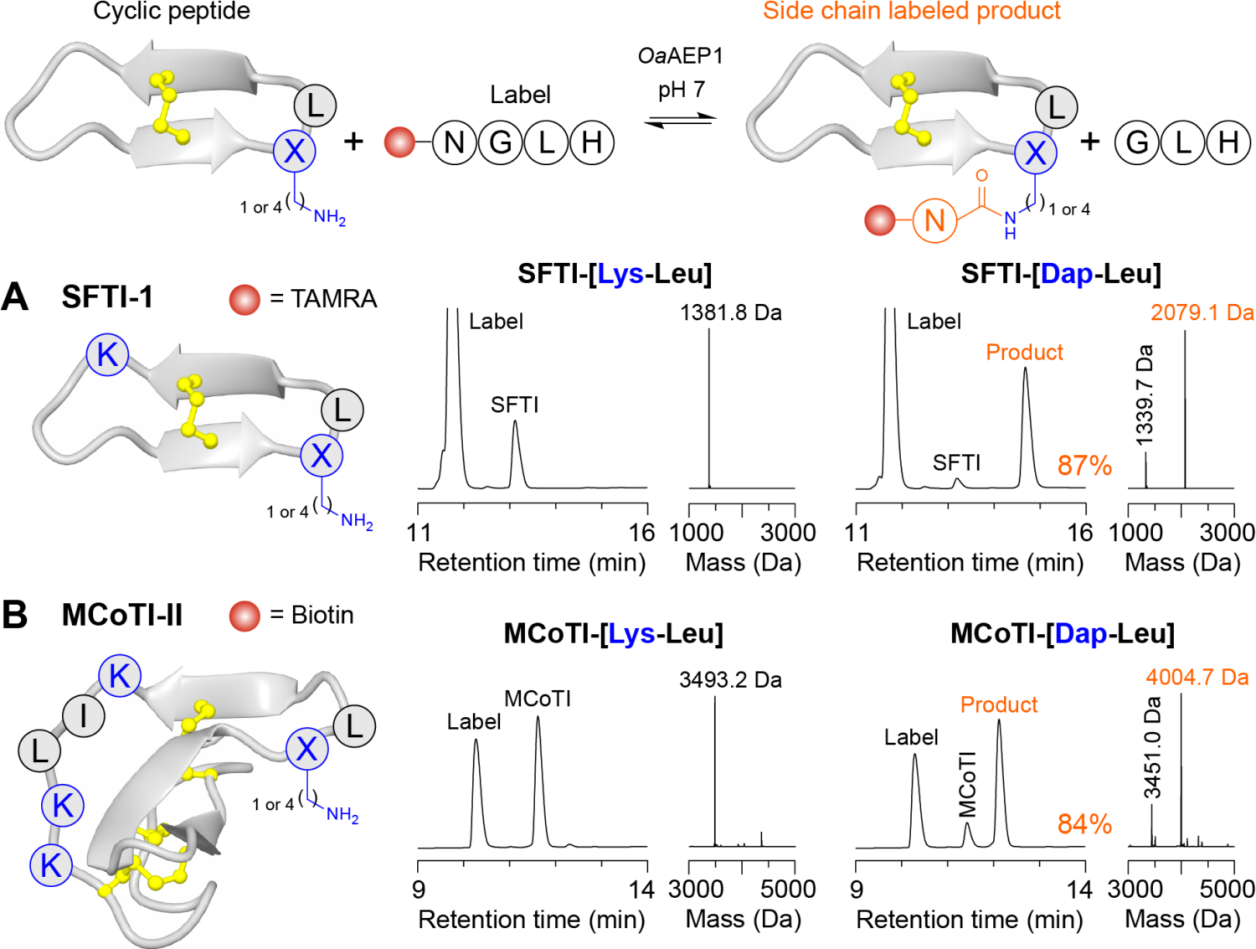
Efficient, site-selective labeling of Lys-containing cyclic peptides via isopeptide ligation at Dap. Labeling of (A) the 14-amino acid cyclic peptide SFTI-1 containing a Lys-Leu or Dap-Leu motif using a TAMRA-GRNGLH peptide, and (B) the 34-amino acid cyclic knottin MCoTI-II containing a Lys-Leu or Dap-Leu motif using a biotin-GRNGLH peptide. Reactions comprised 100 μM cyclic peptide, 400 μM label (4 equiv) and 200 nM *Oa*AEP1 in 100 mM HEPES pH 7 (60 min at 25 °C). Shown are analytical RP-HPLC traces (A214 nm). Peaks for unlabeled cyclic peptide (SFTI or MCoTI), label, and product are indicated. Conversion to product for Dap-Leu analogs was calculated by measuring consumption of the unlabeled cyclic peptide via peak integration at *t* = 0 and 60 min (analytical RP-HPLC traces shown in Figure S20). Reconstructed spectra from ESI-MS of the crude reaction products are also shown, with the observed masses for unlabeled cyclic peptide (black) and product (orange) indicated.

The divergent reactivities of Dap-Leu and Lys-Leu also suggested that *Oa*AEP1-catalyzed isopeptide ligation might enable site-selective labeling at Dap in the presence of Lys residues. To explore this possibility, we turned to labeling of head-to-tail cyclic peptides–this class of substrates lack available termini for conventional transpeptidation reactions, thus are particularly relevant for site-selective side chain modification with *Oa*AEP1. We initially examined the 14-amino acid cyclic peptide sunflower trypsin inhibitor-1 (SFTI-1),^24,25^ which contains a single surface-exposed Lys residue, and synthesized analogs with an embedded Dap-Leu or Lys-Leu tag. Labeling reactions were performed using 100 μM cyclic peptide, 400 μM TAMRA-GRNGLH substrate (4 equiv) and 200 nM *Oa*AEP1 in 100 mM HEPES pH 7 (60 min at 25 °C). The SFTI-1 analog containing a Dap-Leu tag was efficiently labeled under mild conditions, reaching 87% conversion to isopeptide-labeled product (Figures 4A and S20). However, product formation was undetectable for the corresponding analog where the Dap residue was substituted to Lys (Figure 4A). As this peptide contains a Lys-Leu tag and a second Lys residue, the lack of labeled product indicates that labeling of the Dap-containing analog was highly selective at the target Dap-Leu site.

We next examined labeling of the topologically complex cyclic knottin (cyclotide) *Momordica cochinchinensis* trypsin inhibitor-II (MCoTI-II),^26,27^ which comprises 34-amino acids including three disulfide bonds and three endogenous Lys residues. The NMR solution structure of MCoTI-II indicates that two Lys residues (Lys6 and Lys10) are highly solvent-exposed, and the third Lys residue (Lys9) is adjacent to a highly solvent-exposed Leu residue.^28^ We synthesized analogs with an embedded Dap-Leu or Lys-Leu tag and assessed labeling with a biotin-GRNGLH peptide (Figures 4B and S20). As observed for SFTI-1, the MCoTI-II analog with a Dap-Leu tag was efficiently labeled under mild conditions (84% conversion to product). However, only low levels of product were detected (<2%) for the corresponding analog where the Dap-Leu tag was modified to Lys-Leu (Figure 4B). Notably, MCoTI-[Lys-Leu] contains four Lys residues, including separate Lys-Leu and a Leu-Lys sites, further demonstrating that Dap-Leu enables efficient, site-selective modification of cyclic peptides using *Oa*AEP1–an application that is typically challenging with transpeptidases.

**Figure 5:**
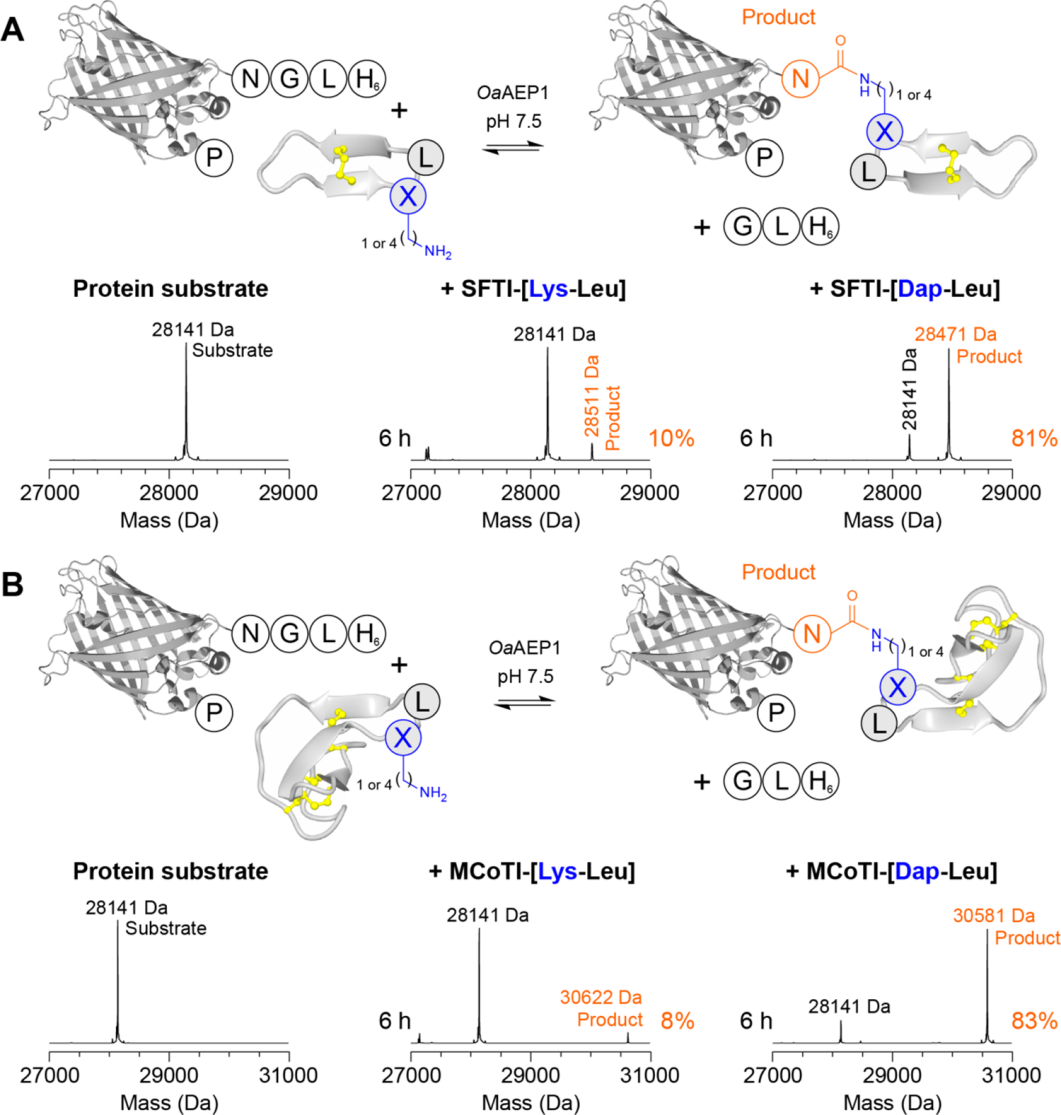
Protein-cyclic peptide fusions via *Oa*AEP1-catalyzed isopeptide ligation. Direct conjugation of (A) the cyclic peptide SFTI-1 containing a Lys-Leu or Dap-Leu motif or (B) the cyclic knottin MCoTI-II containing a Lys-Leu or Dap-Leu motif, to eGFP containing a C-terminal NGL-His_6_ extension. Reactions comprised 25 μM protein, 250 μM cyclic peptide (10 equiv) and 500 nM *Oa*AEP1 in 100 mM HEPES pH 7.5 (reaction time as indicated). Shown are reconstructed spectra from ESI-MS with the observed substrate (black) and product (orange) masses indicated. Additional time points for Dap-containing cyclic peptides are shown in Figure S21.

Finally, we examined whether *Oa*AEP1-catalyzed isopeptide ligation at Dap-Leu could be harnessed for efficient production of protein-cyclic peptide fusions. Such a reaction would enable direct conjugation of a cyclic peptide to a protein with the two molecules linked by a single Asn residue. Initially, we tested ligation of eGFP with a C-terminal NGL-H_6_ extension to SFTI-1 analogs with an embedded Dap-Leu or Lys-Leu tag. In reactions comprising 25 μM protein, 250 μM cyclic peptide (10 equiv) and 500 nM *Oa*AEP1 in 100 mM HEPES pH 7.5 (25 °C), we found that the eGFP-Asn-SFTI fusion was readily formed for the Dap-containing analog, reaching 81% conversion to product after 6 h (Figures 5A and S21). By contrast, only low levels of product (10% conversion) were observed for the Lys-containing analog, which was approximately equivalent to the level of protein substrate hydrolysis (Figure 5A, observed mass: 27,148 Da). We also examined ligation of eGFP to MCoTI-II analogs with an embedded Dap-Leu or Lys-Leu tag to test conjugation of larger, more complex cyclic miniproteins. We observed that the eGFP-Asn-MCoTI fusion was also readily formed (83% conversion after 6 h) for the Dap-containing analog, whereas only minimal product (8% conversion) was detected for the Lys-containing analog (Figures 5B and S21). These findings further broaden the scope of *Oa*AEP1-catalyzed isopeptide ligation at Dap-Leu to include side chain-to-tail conjugation of folded polypeptides.

## Conclusions

In summary, we have developed an efficient approach for site-selective side chain modification using the transpeptidase *Oa*AEP1. Our strategy employs a dipeptide Dap-Leu acceptor that can be embedded within a polypeptide of interest to enable formation of diverse isopeptide-linked products, including engineered peptide topologies and protein–cyclic peptide fusions. These reactions proceed efficiently under mild conditions (pH 7.0–7.5) and do not require cofactors nor quenching of the byproduct released from the acyl donor substrate. Under such conditions, the higher reactivity of Dap compared to its proteinogenic counterpart Lys can be harnessed for highly selective side chain labeling with minimal off-target labeling of Lys residues–a capability that exceeds equivalent chemical approaches. We also demonstrate that *Oa*AEP1-catalyzed isopeptide ligation is compatible with d-peptide acceptors. These reactions occur more readily with a retro-inverso d-Leu-d-Dap motif and further expand the scope of isopeptide-linked products that can be generated, which we exemplify by enzymatically forming a hybrid d-/l- bicyclic peptide topology where both termini are linked to internal side chains.

Transpeptidase-catalyzed side chain modification of peptides and proteins is an emerging application that has significant untapped potential. The ability of *Oa*AEP1 to catalyze isopeptide ligation was only recently identified,^13^ but the reported approach is most productive within narrow constraints that restrict its broader applications. Our study reveals that these limitations can be overcome using Dap, which can be easily incorporated into synthetic peptides during solid-phase synthesis using an inexpensive, commercially available building block. Site-specific incorporation of Dap into ribosomally synthesized proteins is more challenging, which is the main disadvantage of our new approach compared to the previously reported strategy for ligation on Lys residues.^13^ However, an approach that employs genetic code expansion for incorporation of a photoprotected Dap analog has recently been developed.^20,29^ Alternative approaches include total chemical synthesis^30^ or protein semisynthesis,^31^ indicating that isopeptide ligation at Dap could potentially be applied for side chain modification of larger proteins. We anticipate that our strategy for efficient *Oa*AEP1-catalyzed isopeptide ligation will be a valuable addition to established enzymatic approaches for the targeted side chain modification of peptides and proteins.

## Supporting Information Available

Experimental procedures; peptide characterization data; RP-HPLC and MS data supporting additional experiments; NMR spectroscopy (PDF)
