# Supplementary material for: Highly Efficient Transpeptidase-Catalyzed Isopeptide Ligation
Source: J Am Chem Soc. 2024 Dec 23;147(1):557–65. doi: 10.1021/jacs.4c11964 (PMC11726557; doi:10.1021/jacs.4c11964)
Supplement: Supplementary file 1 — ja4c11964_si_001.pdf [file ja4c11964_si_001.pdf]

Supporting Information

Highly Efficient Transpeptidase-Catalyzed Isopeptide Ligation

Simon J. de Veer,*[1] David J. Craik,*[1] Fabian B. H. Rehm*[1,2]

[1]Institute for Molecular Bioscience, Australian Research Council Centre of Excellence for Innovations in Peptide and Protein Science, The University of Queensland, Brisbane, QLD 4072, Australia

[2]Medical Research Council Laboratory of Molecular Biology, Cambridge, UK

Email: s.deveer@imb.uq.edu.au, d.craik@imb.uq.edu.au, fbhrehm@gmail.com

Experimental Section .... Page S2

Supporting Tables .... Page S12

Supporting Figures .... Page S15

1. Experimental Section

Reagents

All reagents were obtained from commercial sources and used as supplied. Solvents for peptide synthesis were from ChemSupply (*N*,*N*-dimethylformamide [DMF], piperidine) or Merck (dichloromethane [DCM], *N*,*N*-diisopropylethylamine [DIPEA]). Fmoc-protected amino acids were from CSBio, except for the following non-canonical amino acids: N-alpha-Fmoc-N-delta-Boc-L-ornithine, N-alpha-Fmoc-N-gamma-Boc-L-2,4-diaminobutyric acid, N-alpha-Fmoc-N-beta-Boc-L-2,3-diaminopropionic acid, N-alpha-Fmoc-N-beta-Boc-D-2,3-diaminopropionic acid, N-chloroacetylglycine (Combi-Blocks), Fmoc-S-trityl-L-homocysteine (AA Blocks), Fmoc-D-proline (Iris Biotech), Fmoc-D-leucine (Mimotopes), Fmoc-D-alanine, Fmoc-D-valine, and Fmoc-O-tert-butyl-D-serine (Chem-Impex). Fmoc-hydrazide was from Chem-Impex. Coupling reagents were from CSBio (O-(1H-6-Chlorobenzotriazole-1-yl)-1,1,3,3-tetramethyluronium hexafluorophosphate, HCTU) or Mimotopes (1-[Bis(dimethylamino)methylene]-1H-1,2,3-triazolo[4,5-b]pyridinium 3-oxid hexafluorophosphate, HATU). Trifluoroacetic acid (TFA) was from ChemSupply and scavengers (thioanisole, triisopropylsilane, 1,2-ethanedithiol) from Merck. Acetonitrile was from Fisher Scientific. D-biotin was from FluoroChem.

Solid-phase peptide synthesis

Peptides were assembled on 2-chlorotrityl chloride resin (CSBio, 0.125 mmol scale, resin loading: 0.7 mmol g^{-1}) using a Symphony multiplex automated synthesizer (Protein Technologies). The dry resin was briefly swelled in DCM, then derivatized with the first Fmoc-amino acid (4 eq.) in DCM:DMF (2:3) containing 8 eq. DIPEA (3 h for Gly, 6 h for Leu or His). Peptide hydrazides were prepared by derivatizing the resin with Fmoc-hydrazide (4 eq.) in DCM:DMF (2:3) containing 8 eq. DIPEA (two reactions: 1 h then 2 h). Unreacted sites were capped using methanol (10% v/v in DMF, 15 min), then the resin was washed 6 times with DMF. Synthesis proceeded via the following protocols, unless stated otherwise:

Deprotection: The N-terminal Fmoc group was removed using 30% (v/v) piperidine in DMF (2 × 3 min), then the resin was washed 6 times with DMF.

Coupling: Solutions of Fmoc-amino acid, HCTU, and DIPEA (all prepared in DMF) were added to the resin and mixed for 10 min (twice). Each repeat was performed using 4 eq. Fmoc-Xaa-OH, 4 eq. HCTU and 8 eq. DIPEA. After the second repeat, the resin was washed 6 times with DMF.

After removing the Fmoc group of the final amino acid, the resin was either:

(i) Washed 3 times with DMF, 2 times with DCM, then dried under N_2.

or

(ii) Washed 4 times with DMF, then the N-terminal amine was acetylated by adding acetic anhydride (20 eq.) and DIPEA (20 eq.) in DMF (30 min). The resin was subsequently washed 3 times with DMF, 2 times with DCM, and dried under N_2.

Cleavage from the resin and side chain deprotection

Peptides were cleaved from the resin and deprotected using 92% TFA, 4% thioanisole, 2% triisopropylsilane, 2% H_2O (3 h). For Cys-rich peptides, 1,2-ethanedithiol (2%) was also included. After removing the resin by filtration, the crude product was precipitated in eight volumes of ice-cold diethyl ether (ChemSupply). The recovered product was dissolved in a mixture of acetonitrile and H_2O then lyophilized.

Peptide purification and validation

Crude peptides were purified by reverse-phase high-performance liquid chromatography (RP-HPLC) using a Shimadzu Prominence system equipped with a Phenomenex Gemini C18 column (5 µm, 110 Å, 250 × 21.2 mm). Briefly, lyophilized peptides were dissolved in a mixture of acetonitrile and H_2O, passed through a 0.45 µm filter, and loaded onto the column. RP-HPLC was performed using a gradient of 5-50% solvent B (90% acetonitrile, 0.1% TFA) in solvent A (0.1% TFA) over 60 min (or similar). Fractions containing the product mass were identified by electrospray ionization mass spectrometry (ESI-MS, Shimadzu Prominence), then pooled and lyophilized. Peptide purity was verified by analytical RP-HPLC (A214 nm) using a Shimadzu Nexera system (Phenomenex Jupiter C18 column, 5 µm, 300 Å, 150 × 2 mm). The table on page S9 lists the peptides synthesized for this study, their calculated mass, and observed mass from matrix-assisted laser desorption ionization time-of-flight mass spectrometry (MALDI-TOF MS) or ESI-MS.

Linear peptides: After lyophilization and validation, purified linear peptides were stored at −20°C until use.

Ac-GWRNGLH

Peptide assembled using the standard protocols detailed above.

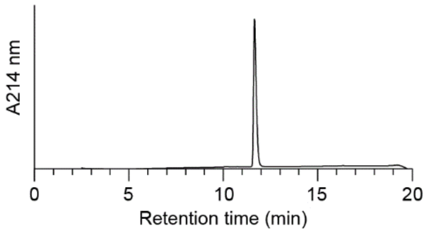

GLRL

Peptide assembled using the standard protocols detailed above.

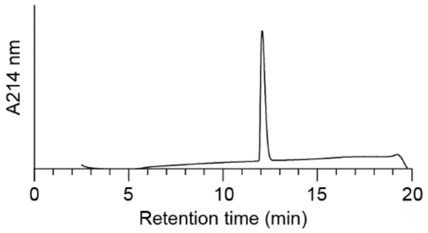

Ac-GKLGV

Peptide assembled using the standard protocols detailed above.

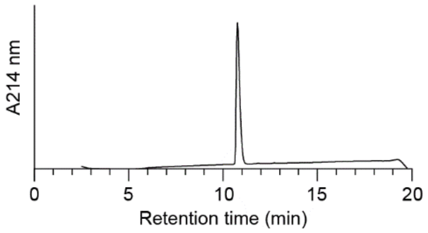

Ac-G[Orn]LGV

Peptide assembled using the standard protocols detailed above and Fmoc-L-Orn(Boc).

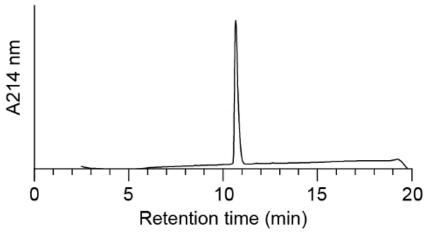

Ac-G[Dab]LGV

Peptide assembled using the standard protocols detailed above and Fmoc-L-Dab(Boc).

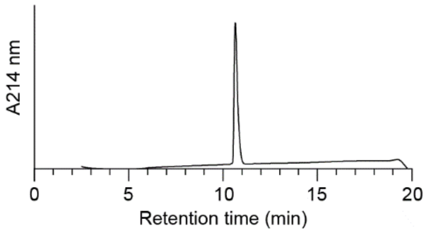

Ac-G[Dap]LGV

Peptide assembled using the standard protocols detailed above and Fmoc-L-Dap(Boc).

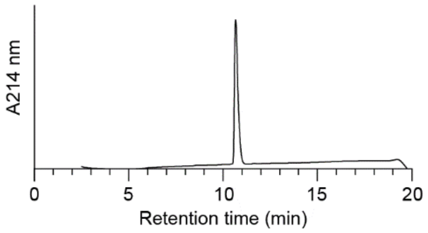

Ac-GLKGV

Peptide assembled using the standard protocols detailed above.

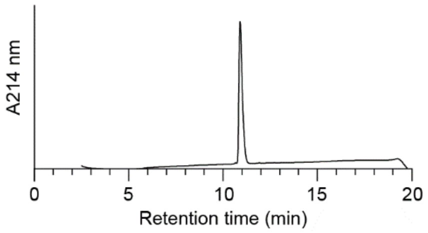

Ac-GL[Orn]GV

Peptide assembled using the standard protocols detailed above and Fmoc-L-Orn(Boc).

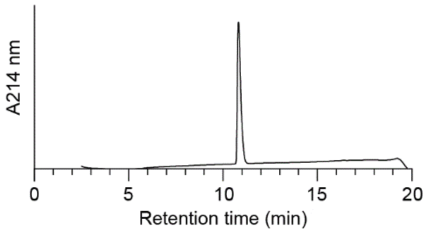

Ac-GL[Dab]GV

Peptide assembled using the standard protocols detailed above and Fmoc-L-Dab(Boc).

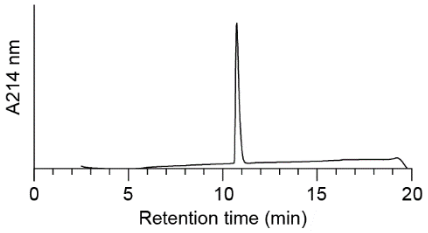

Ac-GL[Dap]GV

Peptide assembled using the standard protocols detailed above and Fmoc-L-Dap(Boc).

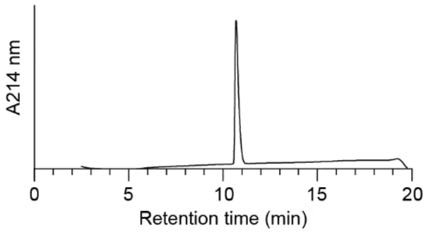

Ac-G[dap]LGV

Peptide assembled using the standard protocols detailed above and Fmoc-D-Dap(Boc).

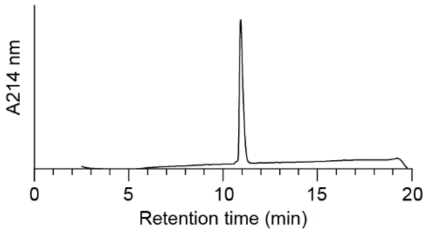

Ac-G[dap]lGv

Peptide assembled using the standard protocols detailed above and Fmoc-D-Dap(Boc), Fmoc-D-Leu and Fmoc-D-Val.

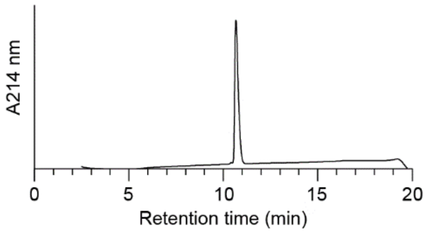

Ac-vGl[dap]G

Peptide assembled using the standard protocols detailed above and Fmoc-D-Val, Fmoc-D-Leu and Fmoc-D-Dap(Boc).

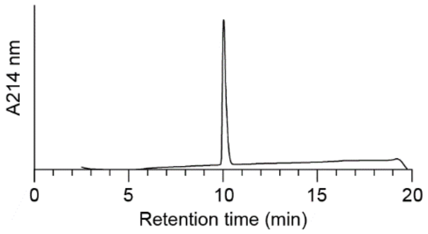

Biotin-GRNGLH

Peptide assembled using the standard protocols detailed above, except that after deprotecting the final amino acid (Gly), an additional coupling reaction was performed using 5 eq. biotin, 4.8 eq. HATU, 10 eq. DIPEA in 1:1 CH_2Cl_2/DMF (5 h), as previously described (Rehm et al. *J. Am. Chem. Soc.* **2019**, *141*, 17388–17393).

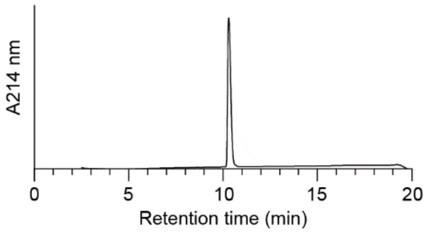

TAMRA-GRNGLH

Peptide assembled using the standard protocols detailed above, except that after deprotecting the final amino acid (Gly), an additional coupling reaction was performed using 3 eq. 5(6)-carboxytetramethylrhodamine (TAMRA, ChemPep), 2.5 eq. HATU, 6 eq. DIPEA in DMF (5 h), as previously described (Rehm et al. *J. Am. Chem. Soc.* **2019**, *141*, 17388–17393).

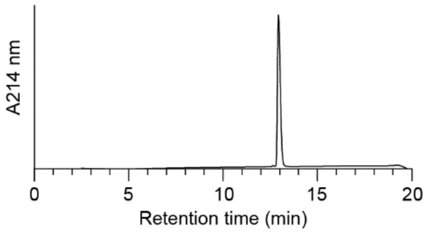

Biotin-G[Dap]LGV

Peptide assembled using the standard protocols detailed above and Fmoc-L-Dap(Boc), except that after deprotecting the final amino acid (Gly), an additional coupling reaction was performed as described for Biotin-GRNGLH.

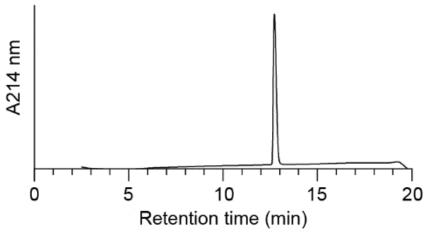

Biotin-vGl[dap]G

Peptide assembled using the standard protocols detailed above and Fmoc-D-Val, Fmoc-D-Leu and Fmoc-D-Dap(Boc), except that after deprotecting the final amino acid (D-Val), an additional coupling reaction was performed as described for Biotin-GRNGLH.

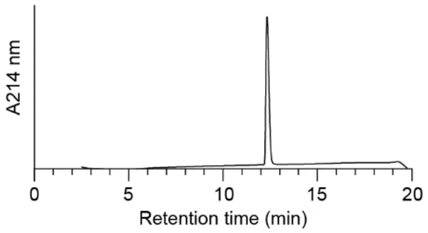

β-hairpin-[Lys-Leu]

Peptide assembled using the standard protocols detailed above and Fmoc-D-Pro.
Sequence: Ac-YGKLTVpGLTRNGLH (p = D-Pro)

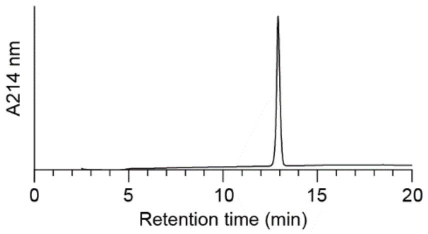

β-hairpin-[Orn-Leu]

Peptide assembled using the standard protocols detailed above and Fmoc-L-Orn(Boc) and Fmoc-D-Pro.
Sequence: Ac-YG[Orn]LTVpGLTRNGLH (p = D-Pro)

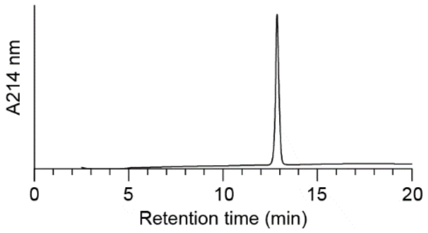

β-hairpin-[Dab-Leu]

Peptide assembled using the standard protocols detailed above and Fmoc-L-Dab(Boc) and Fmoc-D-Pro.
Sequence: Ac-YG[Dab]LTVpGLTRNGLH (p = D-Pro)

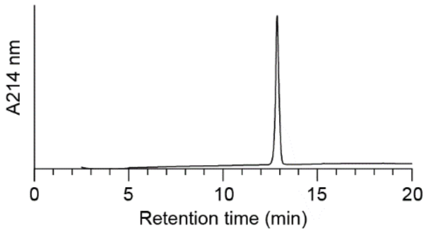

β-hairpin-[Dap-Leu]

Peptide assembled using the standard protocols detailed above and Fmoc-L-Dap(Boc) and Fmoc-D-Pro.
Sequence: Ac-YG[Dap]LTVpGLTRNGLH (p = D-Pro)

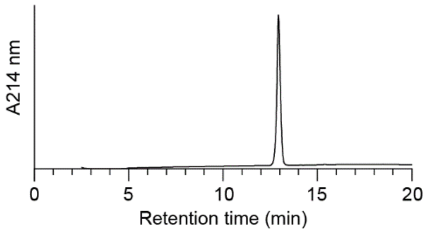

1,3-Dichloroacetone-stapled peptide: Purified linear peptide (0.1 mM) was dissolved in 50 mM NH_4HCO_3 pH 8.1 containing 1.1 eq. tris(2-carboxyethyl)phosphine (TCEP) and incubated at RT (stirring), as previously described (Assem et al. *Angew. Chem. Int. Ed.* **2015**, 54, 8665–8668). After 60 min, 1,3-dichloroacetone (Merck, 1.5 eq. dissolved in DMF) was added and the peptide stapling reaction was incubated for 3 h (RT, stirring). The reaction was acidified, then the stapled product was isolated by RP-HPLC and validated by mass spectrometry and analytical HPLC (as above).
Sequence: Ac-YG[Dap]LGAREA[hCys]ARE[hCys]AAREGNGLH

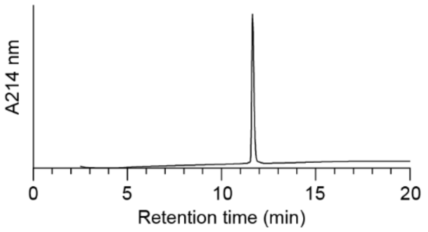

Head-to-side chain cyclic peptides: Purified peptides containing an N-terminal chloroacetyl Gly residue and an internal Cys residue were cyclized via a thioether cross-link. Lyophilized peptide (5 mM) was dissolved in MeCN:H_2O (1:1) containing 2.5% (v/v) DIPEA and stirred for 1 h, as previously described (Johansen-Leete et al. *Chem. Sci.* **2022**, *13*, 3826–3836). The reaction was acidified and diluted ten-fold with H_2O, then the thioether cross-linked product was isolated by RP-HPLC and validated by mass spectrometry and analytical HPLC (as above).

Head-to-side chain cyclic peptide-[Dap-Leu]

Peptide assembled using the standard protocols detailed above and Fmoc-L-Dap(Boc) and N-(chloroacetyl)glycine. Head-to-side chain cyclization was carried out using the protocol above.
Sequence: *c*[GGASG[Dap]LGASGGC]GNGLH

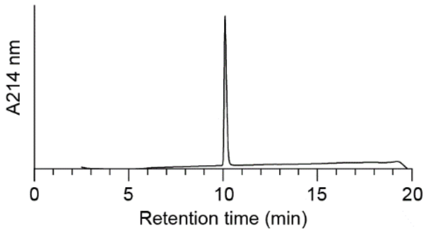

Head-to-side chain cyclic peptide-[leu-dap]

Peptide assembled using the standard protocols detailed above and Fmoc-D-Ala, Fmoc-D-Ser(tBu), Fmoc-D-Dap(Boc) and N-(chloroacetyl)glycine. Head-to-side chain cyclization was carried out using the protocol above.
Sequence: *c*[GGasGl[dap]GasGGC]GNGLH

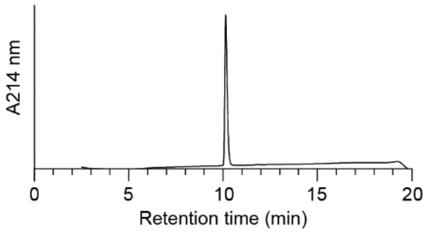

Head-to-tail cyclic peptides: Purified peptide hydrazides were converted to peptide thioesters to enable cyclization via intramolecular native chemical ligation (Fang et al. *Angew. Chem. Int. Ed.* **2011**, *50*, 7645–7649). Lyophilized peptide hydrazides (4 mM) were dissolved in 6 M guanidine HCl pH 3, then the C-terminal hydrazide was activated using 3 eq. acetyl acetone to generate an acyl pyrazole moiety, which subsequently underwent exchange with an aryl thiol, 4-mercaptophenylacetic acid (MPAA, 200 mM), to generate the desired thioester (Flood et al. *Angew. Chem. Int. Ed.* **2018**, *57*, 11634–11639). After 4 h, the reaction was diluted by adding sodium phosphate buffer containing 6 M guanidine HCl and TCEP – final concentration: 100 mM Na_2HPO_4, 6 M guanidine HCl, 50 mM TCEP and 0.5 mM peptide – and the pH was adjusted to 7.0 to facilitate native chemical ligation. After overnight incubation, the reaction was acidified and the head-to-tail cyclic product was purified by RP-HPLC. Finally, disulfide bonds were formed by diluting fractions containing cyclic product in 0.1 M ammonium bicarbonate pH 8.3 (approximately 10 mL per mg of peptide) containing glutathione redox agents and incubated overnight (stirring, RT). After acidifying the reaction, the cyclic disulfide-bonded product was isolated by RP-HPLC, then validated by mass spectrometry and analytical HPLC (as above).

SFTI-[Dap-Leu]

Peptide hydrazide assembled using the standard protocols detailed above and Fmoc-L-Dap(Boc). Cyclization via intramolecular native chemical ligation and disulfide formation were carried out using the protocol above.
Sequence: *c*[GACTKSIPPICA[Dap]L]

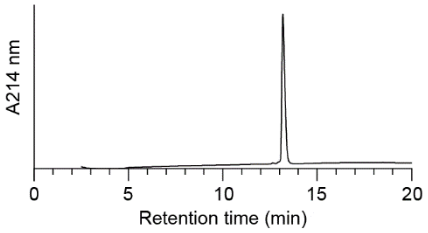

SFTI-[Lys-Leu]

Peptide hydrazide assembled using the standard protocols detailed above. Cyclization via intramolecular native chemical ligation and disulfide formation were carried out using the protocol above.
Sequence: *c*[GACTKSIPPICAKL]

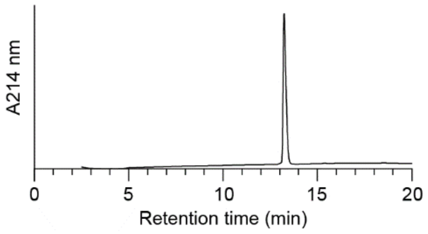

MCoTI-[Dap-Leu]

Peptide hydrazide assembled using the standard protocols detailed above and Fmoc-L-Dap(Boc). Cyclization via intramolecular native chemical ligation and disulfide formation were carried out using the protocol above.
Sequence: *c*[GGVCPKILKKCRRDSDCPGACICRGNGYCGSG[Dap]L]

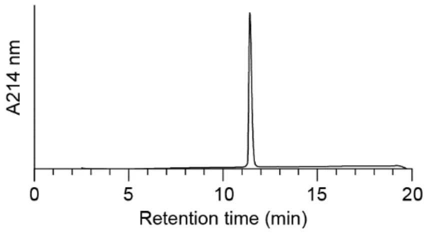

MCoTI-[Lys-Leu]

Peptide hydrazide assembled using the standard protocols detailed above. Cyclization via intramolecular native chemical ligation and disulfide formation were carried out using the protocol above.
Sequence: *c*[GGVCPKILKKCRRDSDCPGACICRGNGYCGSGKL]

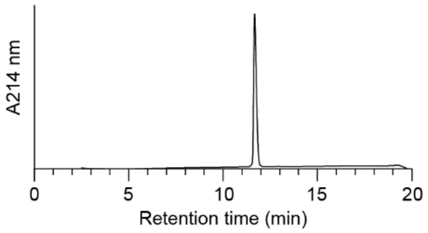

Calculated and observed masses for peptides produced in this study

Peptide	Calculated $[M+H]^+$ (Da)	Observed $[M+H]^+$ (Da)
Ac-GWRNGLH	881.4	881.5
GLRL	458.3	458.3
Ac-GKLGV	515.3	515.4
Ac-G[Orn]LGV	501.3	501.3
Ac-G[Dab]LGV	487.3	487.3
Ac-G[Dap]LGV	473.3	473.3
Ac-GLKGV	515.3	515.4
Ac-GL[Orn]GV	501.3	501.3
Ac-GL[Dab]GV	487.3	487.3
Ac-GL[Dap]GV	473.3	473.3
Ac-G[dap]LGV	473.3	473.3
Ac-G[dap]lGv	473.3	473.3
Ac-vGl[dap]G	473.3	473.3
Biotin-GRNGLH	879.4	879.4
TAMRA-GRNGLH	1065.5	1065.5
Biotin-G[Dap]LGV	657.3	657.4
Biotin-vGl[dap]G	657.3	657.4
β-hairpin-[Lys-Leu]	1667.9	1667.9
β-hairpin-[Orn-Leu]	1653.9	1653.9
β-hairpin-[Dab-Leu]	1639.9	1639.9
β-hairpin-[Dap-Leu]	1625.8	1625.9
Stapled peptide-[Dap-Leu]	2514.2	2514.2
Head-to-side chain cyclic peptide-[Dap-Leu]	1498.6	1498.7
Head-to-side chain cyclic peptide-[leu-dap]	1498.6	1498.7
SFTI-[Dap-Leu]	1339.7	1339.7
SFTI-[Lys-Leu]	1381.8	1381.8
MCoTI-[Dap-Leu]	3451.1	3451.0
MCoTI-[Lys-Leu]	3493.2	3493.2

Recombinant protein production

Proteins were produced recombinantly in *E. coli* BL21 (P-eGFP-NGL-6H) or SHuffle ([C247A]*Oa*AEP1). Plasmids (pET14b for eGFP or pHUE for [C247A]*Oa*AEP1) were transformed into the appropriate strain and expression was induced via IPTG addition to 0.3–0.4 mM after cultures (30°C for SHuffle, 37°C for BL21) reached an OD_{600} of 0.6–0.8. Subsequently, cells were harvested by centrifugation and resuspended in lysis buffer (50 mM NaH_2PO_4, 300 mM NaCl, 10 mM imidazole, pH 8), lyzed using a Constant Systems cell disruptor (32 kpsi), and clarified via centrifugation. Next, the His-tagged proteins were purified via NiNTA (Qiagen), following the manufacturer's guidelines. The P-eGFP-NGL-6H substrate was further purified via size exclusion chromatography on a column (HiLoad 16/600 Superdex 75 pg) equilibrated with PBS. For *Oa*AEP1, we purified the enzyme further via size exclusion chromatography (HiLoad 16/600 Superdex 75 pg, equilibrated with 20 mM HEPES pH 8, 100 mM NaCl, 10% glycerol), combined and concentrated the fractions, and activated the enzyme via addition of acetic acid to pH 4.0–4.5 and incubation at 37°C for 2–4 h (monitored by SDS-PAGE). Protein concentrations were determined via NanoDrop A_{280} readings using calculated protein extinction coefficients and molecular weights. All proteins were stored at −80°C until use.

Intermolecular peptide ligation reactions

Reactions for ligation of amine-containing peptide nucleophiles (500 µM) to a model NGLH-containing peptide substrate (Ac-GWRNGLH, 100 µM) were run using 200 nM [C247A]*Oa*AEP1 with or without $NiCl_2$ (300 µM) in 100 mM HEPES pH 7.5 at 25°C until they were quenched via TFA addition to 1% (v/v). The quenched solutions were analyzed by RP-HPLC using a Shimadzu Nexera system (Phenomenex Jupiter C18 column, 5 µm, 300 Å, 150 × 2 mm, 1–50% acetonitrile gradient) and MALDI-TOF MS (SCIEX TOF/TOF 5800 MALDI mass spectrometer, α-cyano-4-hydroxycinnamic acid matrix). Percent conversion to product was calculated based on peak integration from analytical RP-HPLC. Reactions were repeated three times.

Peptide side chain-to-tail cyclization

Reactions for intramolecular side chain-to-tail cyclization of β-hairpin peptides (sequence: Ac-YGXLTVpGLTRNGLH, p = D-Pro and X = Lys, Orn, Dab or Dap) were carried out using 50 µM peptide and 100 nM *Oa*AEP1 in 100 mM HEPES pH 7.5 at 25°C until they were quenched via TFA addition to 1% (v/v). Reactions for intramolecular side chain-to-tail cyclization of the stapled helical peptide were carried out using 50 µM substrate and 200 nM *Oa*AEP1 in 100 mM HEPES pH 7.5 at 25°C until they were quenched via TFA addition to 1% (v/v). The quenched solutions were analyzed by RP-HPLC using a Shimadzu Nexera system (Phenomenex Jupiter C18 column, 5 µm, 300 Å, 150 × 2 mm, 1–50% acetonitrile gradient) and MALDI-TOF MS (SCIEX TOF/TOF 5800 MALDI mass spectrometer, α-cyano-4-hydroxycinnamic acid matrix). Percent conversion to product was calculated based on peak integration from analytical RP-HPLC. Reactions were repeated three times.

Reactions for *Oa*AEP1-catalyzed formation of dual cross-linked, bicyclic peptides were carried out using purified thioether cross-linked substrates (50 µM) and 200 nM *Oa*AEP1 in 100 mM HEPES pH 7.5 at 25°C until they were quenched via TFA addition to 1% (v/v). The quenched solutions were analyzed by RP-HPLC using a Shimadzu Nexera system (Phenomenex Jupiter C18 column, 5 µm, 300 Å, 150 × 2 mm, 1–50% acetonitrile gradient) and ESI-MS (SCIEX 5600 TripleTOF electrospray ionization mass spectrometer – full details provided in the protein labeling section). Reconstructed spectra were generated using MagTran software (Amgen). Percent conversion to product was calculated based on peak integration from analytical RP-HPLC. Reactions were repeated three times.

Cyclic peptide labeling

Reactions for side chain labeling of SFTI-1 or MCoTI-II analogs with an embedded Dap-Leu or Lys-Leu tag were carried out using 100 µM cyclic peptide, 400 µM label (TAMRA-GRNGLH for SFTI-1, biotin-GRNGLH for MCoTI-II; 4 equiv.) and 200 nM *Oa*AEP1 in 100 mM HEPES pH 7 at 25 °C until they were quenched via TFA addition to 1% (v/v). The quenched solutions were analyzed by RP-HPLC using a Shimadzu Nexera system (Phenomenex Jupiter C18 column, 5 µm, 300 Å, 150 × 2 mm, 1–50% acetonitrile gradient) and ESI-MS (SCIEX 5600 TripleTOF electrospray ionization mass spectrometer – full details provided in the protein labeling section). Reconstructed spectra were generated using MagTran (Amgen) or Analyst (SCIEX) software. Percent conversion to product was calculated by measuring consumption of the unlabeled cyclic peptide via peak integration from analytical RP-HPLC for samples quenched at t = 0 min and 60 min. Reactions were repeated three times.

Protein labeling reactions

Protein labeling reactions were carried out using 25 µM P-eGFP-NGL-6H substrate, 250 µM peptide (biotin-G[Dap]LGV, biotin-vGl[D-Dap]G, SFTI-1 analogs or MCoTI-II analogs) and 500 nM *Oa*AEP1 in 100 mM HEPES pH 7.5 at 25°C until they were quenched via TFA addition to 1% (v/v). The quenched solutions were loaded onto a Zorbax 300SBC18 column (Agilent) and eluted over a 15 min 1–50% acetonitrile gradient. The column outflow was directed to a 5600 TripleTOF (SCIEX) electrospray ionization mass spectrometer. Reconstructed spectra were generated using Analyst software (SCIEX). Percent conversion to product was calculated based on relative peak areas determined using Analyst. Reactions were repeated three times.

NMR spectroscopy

Purified peptide substrates or isopeptide-linked products (~1 mg) were dissolved in 550 µL H_2O/D_2O (10:1) at pH 3.5–4, and 4,4-dimethyl-4-silapentane-1-sulfonic acid (DSS) was added as an internal standard (referenced to 0.00 ppm). Peptides were analyzed by performing ^{1}H 1D, ^{1}H–^{1}H total correlation spectroscopy (TOCSY), and ^{1}H–^{1}H nuclear Overhauser effect spectroscopy (NOESY) experiments, which were conducted at 298 K using a Bruker AVANCE 600 MHz NMR spectrometer equipped with a cryogenically cooled probe. For TOCSY and NOESY experiments, mixing times were 80 and 200 ms, respectively. Spectra were assigned in CCPNMR 2.2.2 using the sequential assignment protocol. Secondary Hα chemical shifts were calculated using previously reported random coil shifts (Wishart et al. *J. Biomol. NMR* **1995**, 5, 67–81) and the equation: secondary chemical shift (Δδ) = δ(observed) – δ(random coil).

2. Supporting Tables

Table S1. Monoisotopic masses for isopeptide ligation products from reactions using peptide nucleophiles bearing a side chain amine and a model acyl donor substrate (Ac-GWRNGLH).

	Calculated $[M+H]^+$ (Da)	Observed $[M+H]^+$ (Da)
Figure S1, S4		
Acyl donor substrate (Ac-GWRNGLH)	881.4	881.5
Hydrolyzed substrate (Ac-GWRN)	574.3	574.4
Ac-GKLGV product	1070.6	1070.6
Ac-G[Orn]LGV product	1056.6	1056.6
Ac-G[Dab]LGV product	1042.5	1042.5
Ac-G[Dap]LGV product	1028.5	1028.5
Ac-GLKGV product	1070.6	1070.6
Ac-GL[Orn]GV product	1056.6	1056.6
Ac-GL[Dab]GV product	1042.5	1042.5
Ac-GL[Dap]GV product	1028.5	1028.5
Ac-G[dap]LGV product (D-Dap)	1028.5	1028.6
Ac-G[dap]lGv product (mirror image)	1028.5	1028.5
Ac-vGl[dap]G product (retro-inverso)	1028.5	1028.6
Figure S8		
Ac-AG[Dap]LGA product	1071.6	1071.5
Ac-AG[Dap]IGA product	1071.6	1071.5
Ac-AG[Dap]FGA product	1105.6	1105.5
Ac-AG[Dap]GGA product	1015.5	1015.5
Ac-AG[Dap]PGA product	1055.5	1055.5
Ac-AG[Dap]QGA product	1086.5	1086.5
Ac-AG[Dap]SGA product	1045.5	1045.5
Figure S9		
Ac-AI[Dap]LGA product	1127.6	1127.6
Ac-AF[Dap]LGA product	1161.6	1161.5
Ac-AG[Dap]LGA product	1071.6	1071.5
Ac-AP[Dap]LGA product	1111.6	1111.5
Ac-AQ[Dap]LGA product	1142.6	1142.6
Ac-AS[Dap]LGA product	1101.6	1101.5

Table S2. Masses for isopeptide cyclization products and side chain-labeled cyclic peptides

	Calculated $[M+H]^+$ (Da)	Observed $[M+H]^+$ (Da)
Figure S13		
β-hairpin-[Lys-Leu]	1342.8	1342.8
β-hairpin-[Orn-Leu]	1328.7	1328.8
β-hairpin-[Dab-Leu]	1314.7	1314.8
β-hairpin-[Dap-Leu]	1300.7	1300.7
Figure S16		
Stapled peptide-[Dap-Leu]	2189.0	2189.1
Figure 2B, S17		
Bicyclic peptide-[Dap-Leu]	1173.4	1173.4
D-/L- bicyclic peptide-[leu-dap]	1173.4	1173.3
Figure 4		
SFTI-[Dap-Leu]-(TAMRA-GRN)	2079.0	2079.1
SFTI-[Lys-Leu]-(TAMRA-GRN)	2121.1	N.D.
MCoTI-[Dap-Leu]-(biotin-GRN)	4004.8	4004.7
MCoTI-[Lys-Leu]-(biotin-GRN)	4046.9	N.D.

N.D. = not detected

Table S3. Average masses for isopeptide-linked protein-peptide fusion products

	Calculated mass (Da)	Observed mass (Da)
P-eGFP-NGL-6H	28,139	28,141
Figure 1C		
P-eGFP-N-(biotin-G[Dap]LGV)	27,786	27,788
P-eGFP-N-(biotin-vGl[dap]G)	27,786	27,788
Figure 5		
P-eGFP-N-(SFTI-[Dap-Leu])	28,468	28,471
P-eGFP-N-(SFTI-[Lys-Leu])	28,510	28,511
P-eGFP-N-(MCoTI-[Dap-Leu])	30,579	30,581
P-eGFP-N-(MCoTI-[Lys-Leu])	30,621	30,622

P-eGFP-NGL-6H sequence
(final substrate underlined, chromophore formation results in a mass shift of –20 Da)

MPVSKGEELFTGVVPILVELDGDVNGHKFSVSGEGEGDATYGKLTLKFICTTGKLPVPWPTLVTTLTYGVQCFSRYPD
HMKQHDFFKSAMPEGYVQERTIFFKDDGNYKTRAEVKFEGDTLVNRIELKGIDFKEDGNILGHKLEYNYNSHNVYI
MADKQKNGIKVNFKIRHNIEDGSVQLADHYQQNTPIGDGPVLLPDNHYLSTQSALSKDPNEKRDHMVLLEFVTAA
GITLGMDELYKGSNGLHHHHHH

3. Supporting Figures

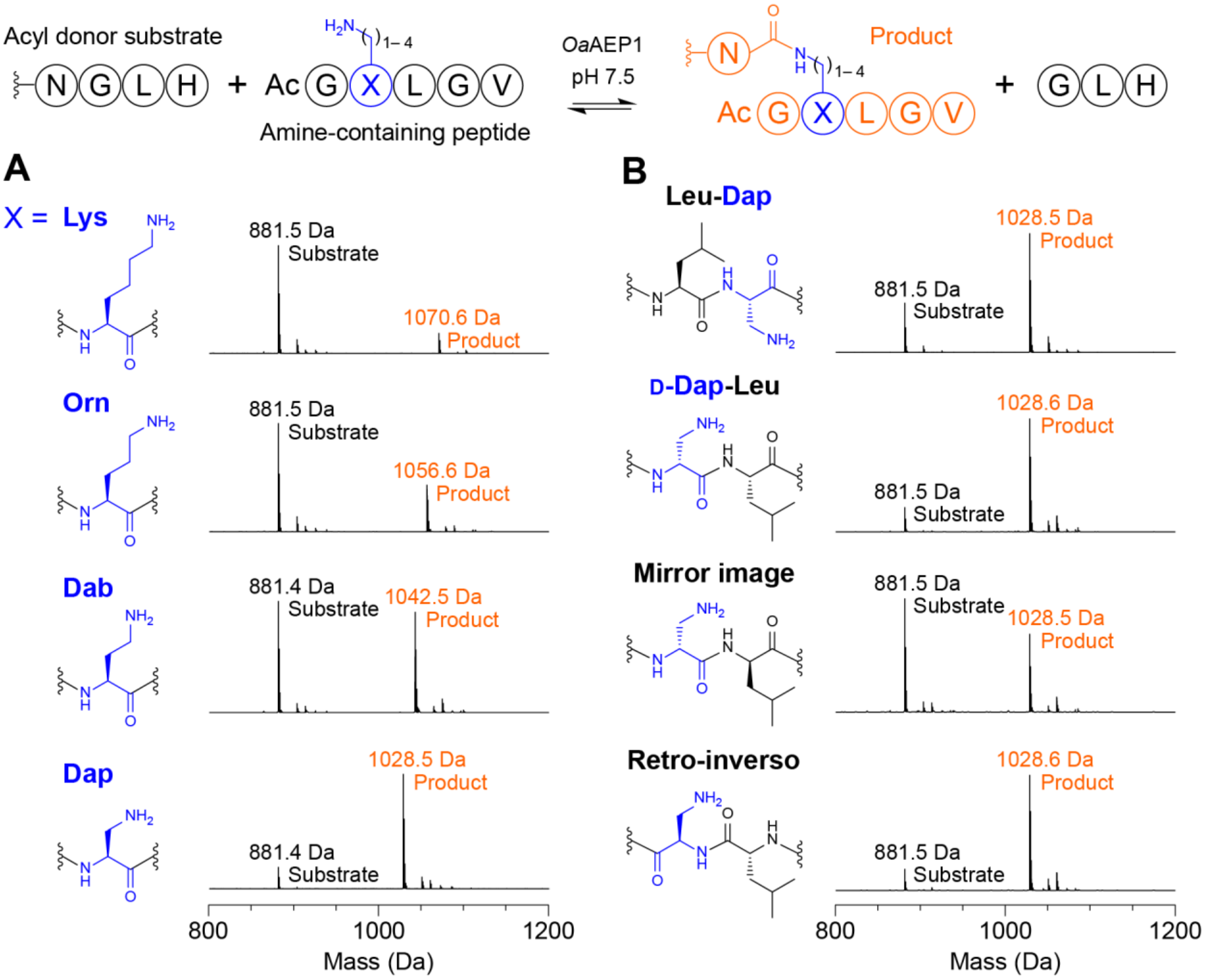

Figure S1. MALDI-TOF MS spectra for asparaginyl ligase-catalyzed isopeptide ligation at near-neutral pH. (A) Nα-acetylated peptides bearing a side chain amine (Ac-GXLGV where X = Lys, ornithine (Orn), 2,4-diaminobutyric acid (Dab) or 2,3-diaminopropionic acid (Dap), 500 µM, 5 equiv.) were ligated to an NGLH-containing substrate (Ac-GWRNGLH, 100 µM) using 200 nM *Oa*AEP1 in 100 mM HEPES pH 7.5 (45 min at 25 °C). Peaks for acyl donor substrate (black) and product (orange) are indicated, as well as the observed mass of each species. Analytical RP-HPLC traces are shown in Figure 1A. (B) Isopeptide ligation with modified Dap substrates. Reactions were carried out as in (A) using Ac-GL[Dap]GV (Leu-Dap), Ac-G[D-Dap]LGV (D-Dap-Leu), Ac-G[D-Dap]lGv (mirror image) or Ac-vGl[D-Dap]G (retro-inverso). Analytical RP-HPLC traces are shown in Figure 1B.

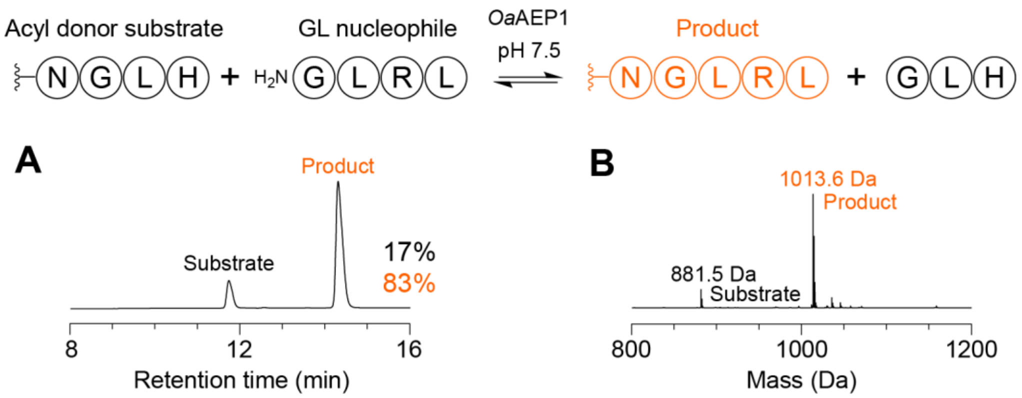

Figure S2. Product formation for a conventional transpeptidation reaction with an optimal GL nucleophile. Reactions were carried out as in Figure 1A using 500 µM GLRL, 100 µM acyl donor substrate (Ac-GWRNGLH) and 200 nM *Oa*AEP1 in 100 mM HEPES pH 7.5 (45 min at 25 °C). (A) Analytical RP-HPLC trace (A280 nm) with peaks for acyl donor substrate (black) and product (orange) indicated, as well as the percentage of each species. (B) Spectrum from MALDI-TOF MS with peaks for acyl donor substrate (black) and product (orange) indicated, as well as the observed mass of each species.

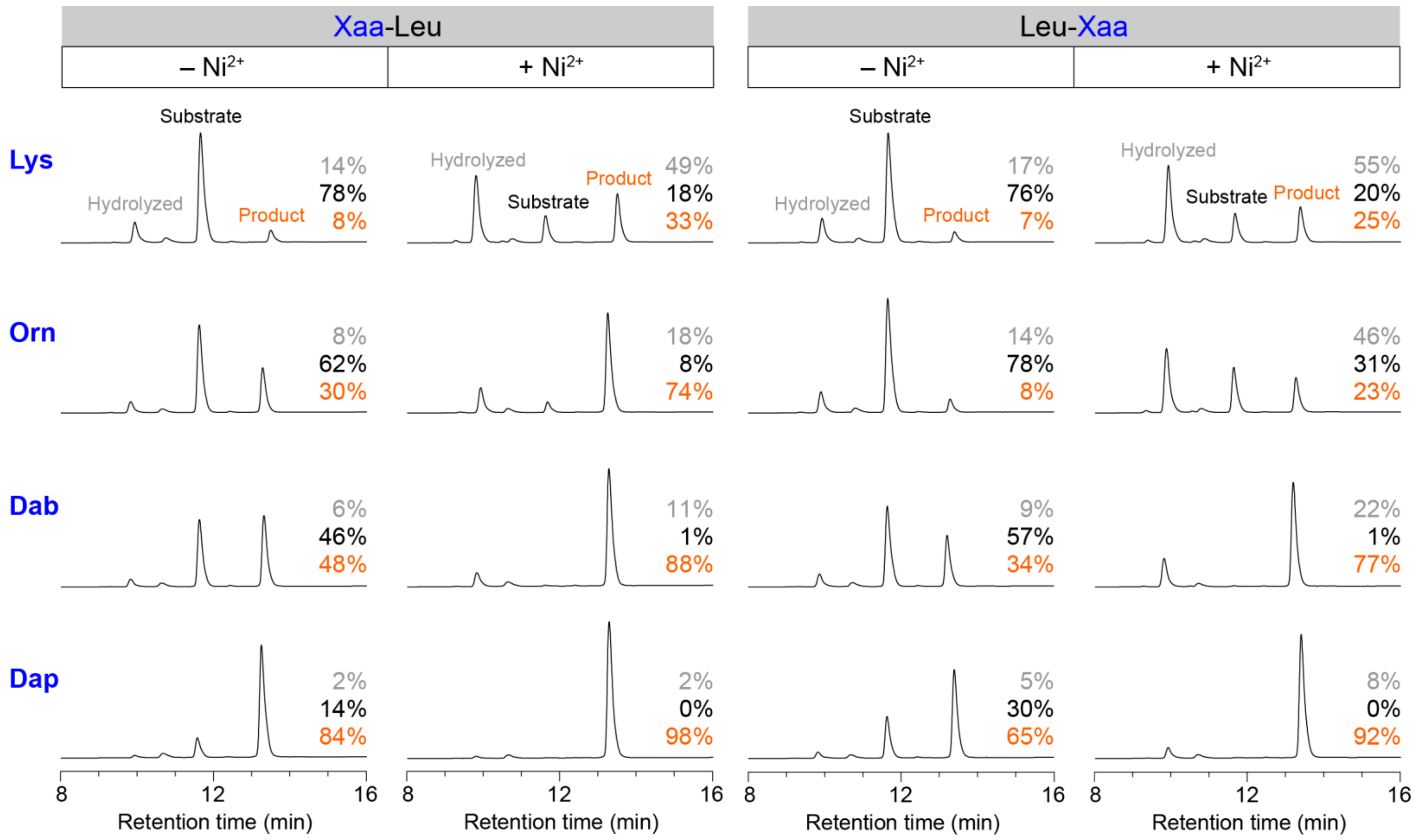

Figure S3. Expanded reaction series for asparaginyl ligase-catalyzed isopeptide ligation at near-neutral pH. Nα-acetylated peptides bearing a side chain amine (Ac-GXLGV or Ac-GLXGV, where X = Lys, ornithine (Orn), 2,4-diaminobutyric acid (Dab) or 2,3-diaminopropionic acid (Dap), 500 µM, 5 equiv.) were ligated to an NGLH-containing substrate (Ac-GWRNGLH, 100 µM) using 200 nM *Oa*AEP1 in 100 mM HEPES pH 7.5 (45 min at 25 °C). Additional reactions were run with 300 µM $NiCl_2$ (indicated by + Ni^{2+}) to quench the GLH byproduct released upon cleavage of the acyl donor substrate. Shown are analytical RP-HPLC traces (A280 nm). Peaks for acyl donor substrate (black), product (orange), and hydrolyzed substrate (grey) are indicated, as well as the percentage of each species. Spectra from MALDI-TOF MS are shown in Figure S4.

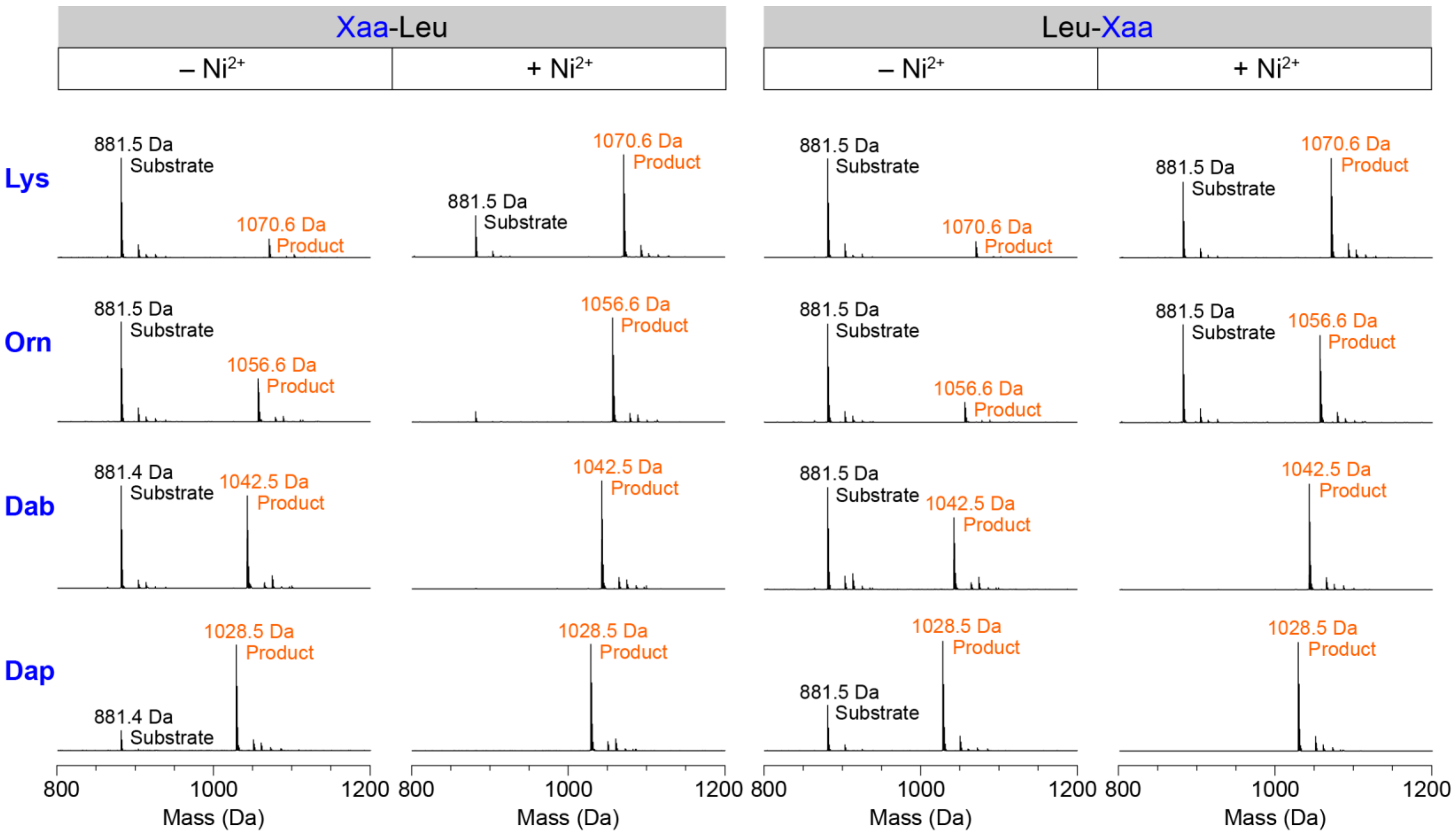

Figure S4. MALDI-TOF MS spectra for expanded reaction series for asparaginyl ligase-catalyzed isopeptide ligation at near-neutral pH. Nα-acetylated peptides bearing a side chain amine (Ac-GXLGV or Ac-GLXGV, where X = Lys, ornithine (Orn), 2,4-diaminobutyric acid (Dab) or 2,3-diaminopropionic acid (Dap), 500 µM, 5 equiv.) were ligated to an NGLH-containing substrate (Ac-GWRNGLH, 100 µM) using 200 nM *Oa*AEP1 in 100 mM HEPES pH 7.5 (45 min at 25 °C). Additional reactions were run with 300 µM $NiCl_2$ (indicated by + Ni^{2+}) to quench the GLH byproduct released upon cleavage of the acyl donor substrate. Peaks for acyl donor substrate (black) and product (orange) are indicated, as well as the observed mass of each species. Analytical RP-HPLC traces are shown in Figure S3.

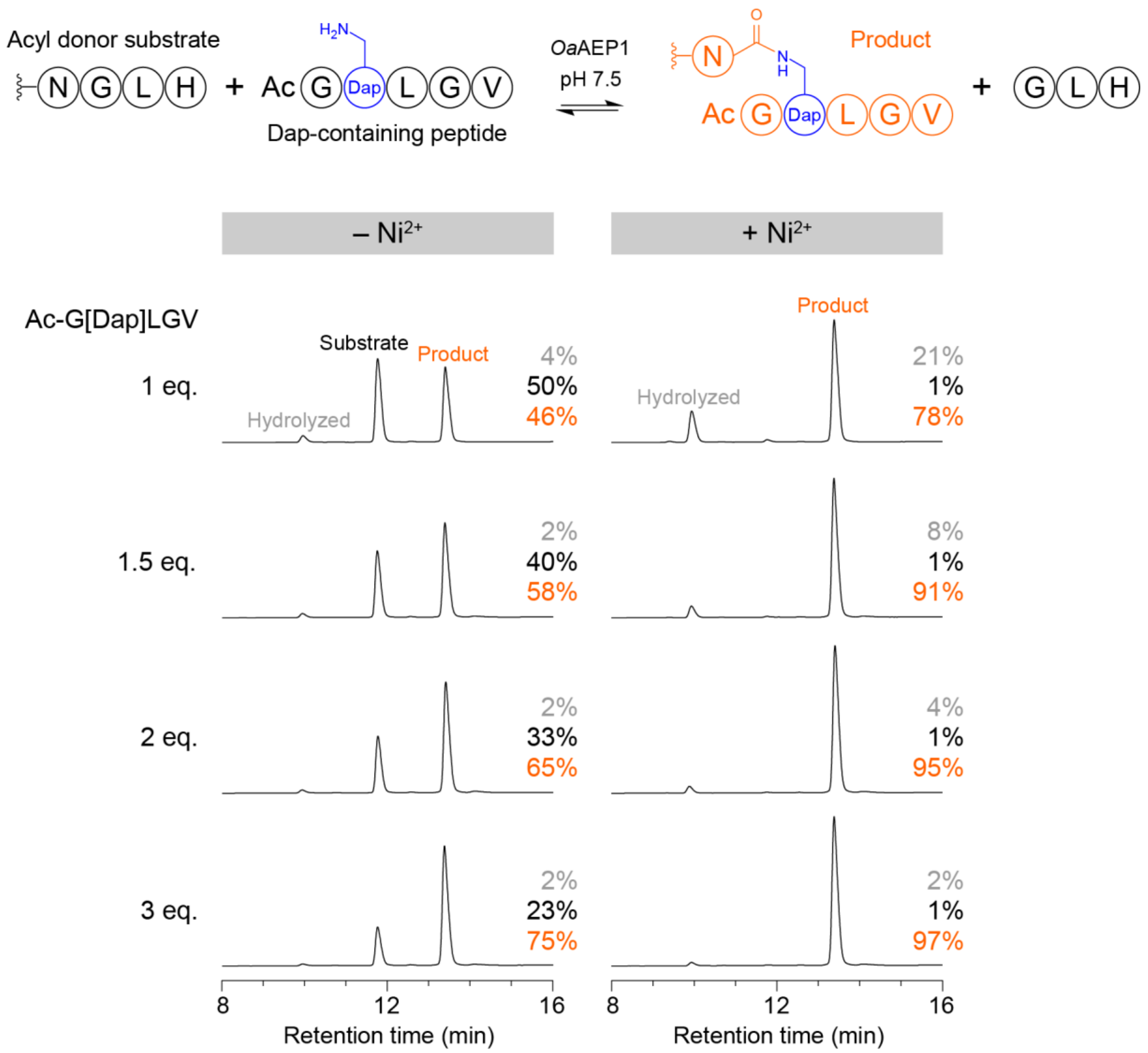

Figure S5. Asparaginyl ligase-catalyzed isopeptide ligation with varying concentrations of Dap-acceptor peptide. Ac-G[Dap]LGV (100–300 µM, 1–3 equiv. relative to the acyl donor substrate, as indicated) was ligated to an NGLH-containing substrate (Ac-GWRNGLH, 100 µM) using 200 nM *Oa*AEP1 in 100 mM HEPES pH 7.5 (45 min at 25 °C). Additional reactions were run with 300 µM $NiCl_2$ (indicated by + Ni^{2+}) to quench the GLH byproduct released upon cleavage of the acyl donor substrate. Shown are analytical RP-HPLC traces (A280 nm). Peaks for acyl donor substrate (black), product (orange), and hydrolyzed substrate (grey) are indicated, as well as the percentage of each species. Spectra from MALDI-TOF MS are shown in Figure S6.

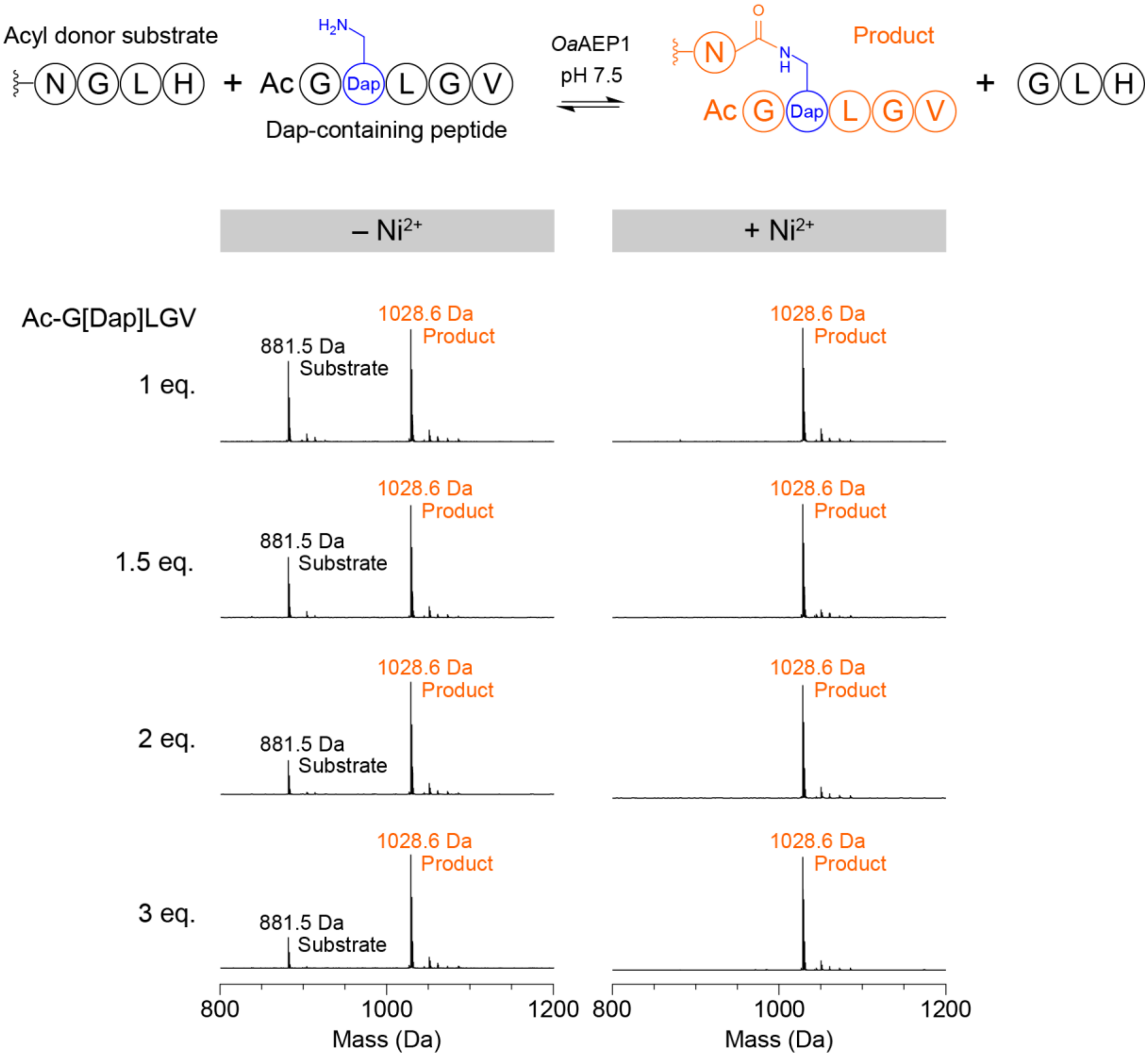

Figure S6. MALDI-TOF MS spectra for asparaginyl ligase-catalyzed isopeptide ligation with varying concentrations of Dap-acceptor peptide. Ac-G[Dap]LGV (100–300 µM, 1–3 equiv. relative to the acyl donor substrate, as indicated) was ligated to an NGLH-containing substrate (Ac-GWRNGLH, 100 µM) using 200 nM *Oa*AEP1 in 100 mM HEPES pH 7.5 (45 min at 25 °C). Additional reactions were run with 300 µM $NiCl_2$ (indicated by + Ni^{2+}) to quench the GLH byproduct released upon cleavage of the acyl donor substrate. Peaks for acyl donor substrate (black) and product (orange) are indicated, as well as the observed mass of each species. Analytical RP-HPLC traces are shown in Figure S5.

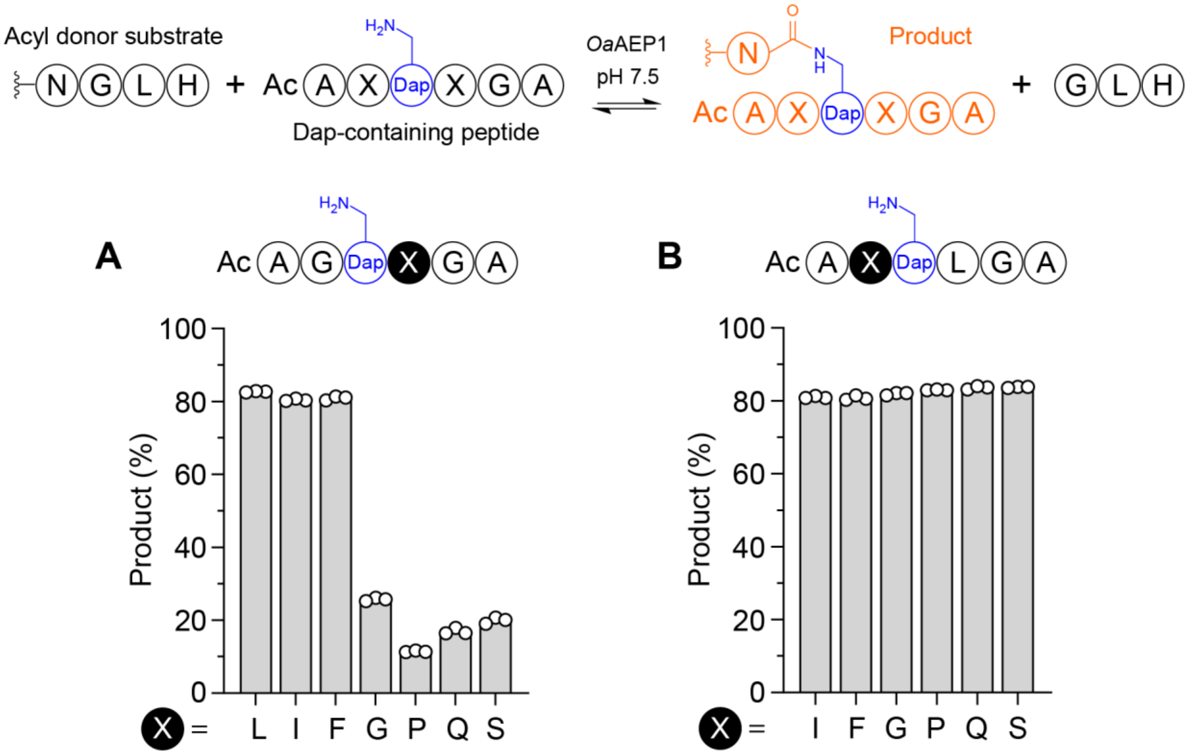

Figure S7. Substrate scope for asparaginyl ligase-catalyzed isopeptide ligation at Dap-Xaa or Xaa-Dap. Nα-acetylated peptides (Ac-AG[Dap]XGA or Ac-AX[Dap]LGA, 500 µM, 5 equiv.) were ligated to an NGLH-containing substrate (Ac-GWRNGLH, 100 µM) using 200 nM *Oa*AEP1 in 100 mM HEPES pH 7.5 (45 min at 25 °C). Conversion to product was determined by peak integration from analytical RP-HPLC (A280 nm), and product identity was verified by MALDI-TOF MS (Figure S8–S9). Bar graphs illustrate the mean conversion to product (%) from three experiments (individual data points shown), and the variable residue (X) is indicated on the *x*-axis.

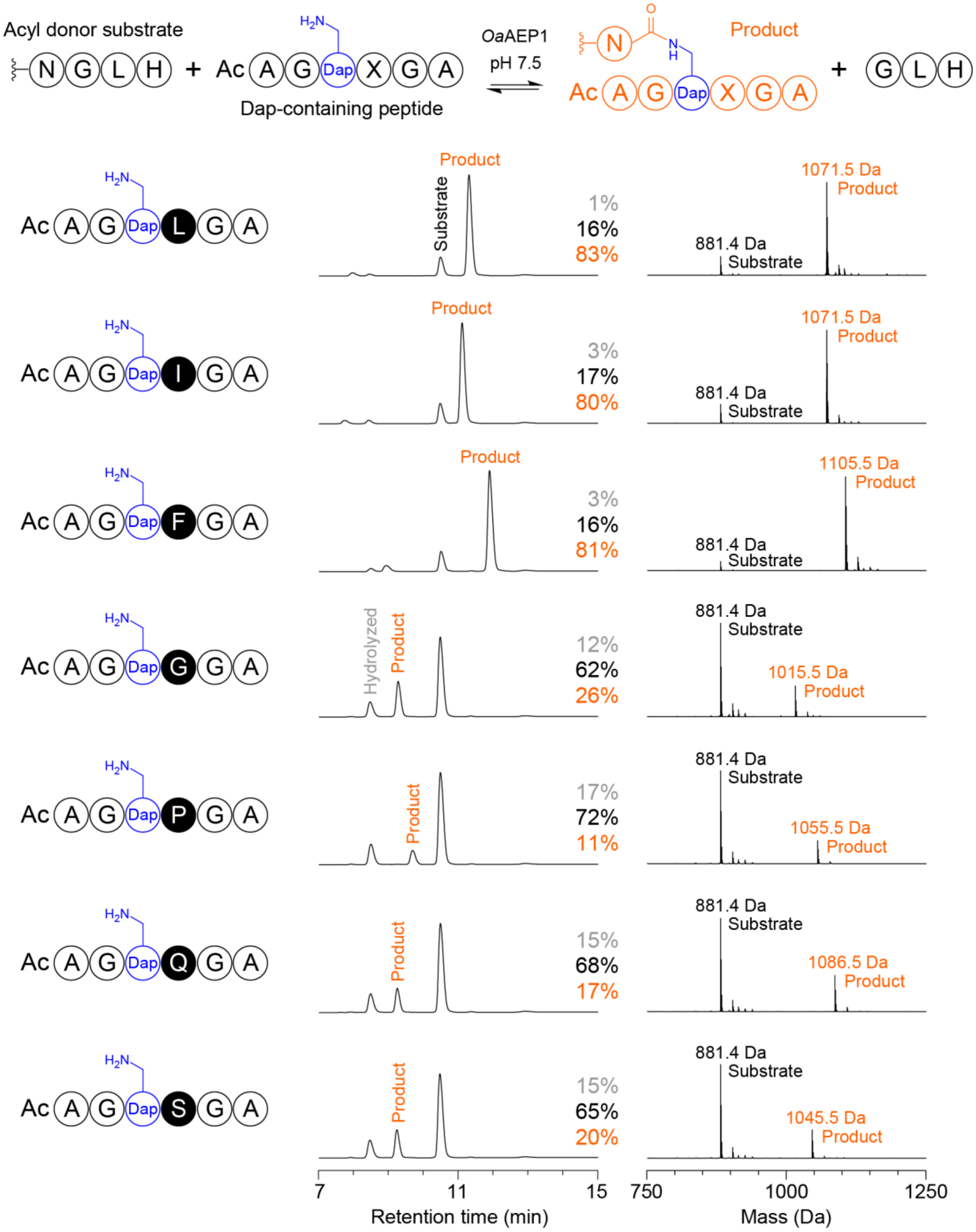

Figure S8. Substrate scope for asparaginyl ligase-catalyzed isopeptide ligation at Dap-Xaa. Nα-acetylated peptides (Ac-AG[Dap]XGA, where X = Leu, Ile, Phe, Gly, Pro, Gln or Ser, 500 µM, 5 equiv.) were ligated to an NGLH-containing substrate (Ac-GWRNGLH, 100 µM) using 200 nM *Oa*AEP1 in 100 mM HEPES pH 7.5 (45 min at 25 °C). Left panels show analytical RP-HPLC traces (A280 nm) with peaks for acyl donor substrate (black), product (orange), and hydrolyzed substrate (grey) indicated, as well as the percentage of each species. Right panels show spectra from MALDI-TOF MS with peaks for acyl donor substrate (black) and product (orange) indicated, as well as the observed mass of each species.

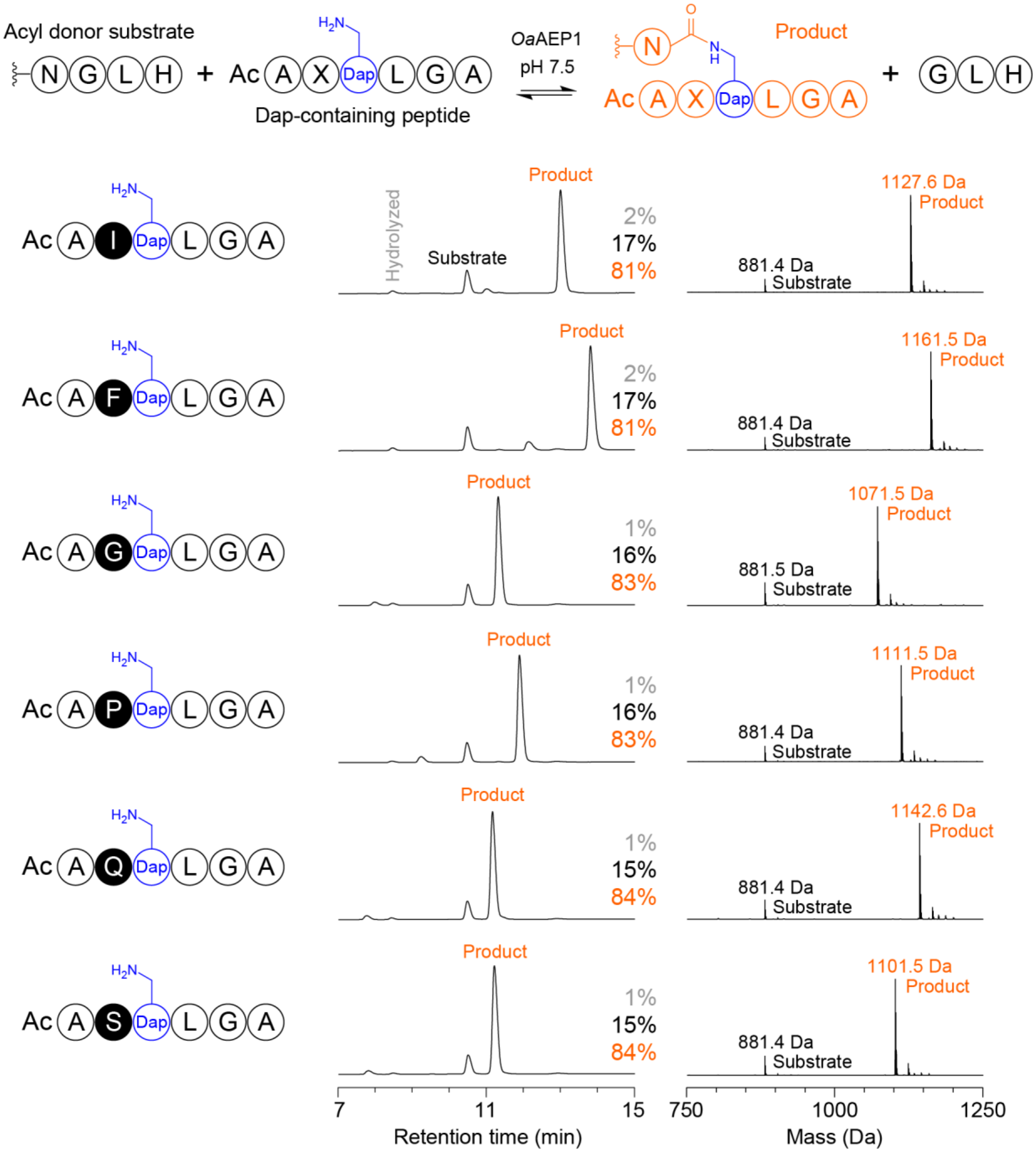

Figure S9. Substrate scope for asparaginyl ligase-catalyzed isopeptide ligation at Xaa-Dap. Nα-acetylated peptides (Ac-AX[Dap]LGA, where X = Ile, Phe, Gly, Pro, Gln or Ser, 500 µM, 5 equiv.) were ligated to an NGLH-containing substrate (Ac-GWRNGLH, 100 µM) using 200 nM *Oa*AEP1 in 100 mM HEPES pH 7.5 (45 min at 25 °C). Left panels show analytical RP-HPLC traces (A280 nm) with peaks for acyl donor substrate (black), product (orange), and hydrolyzed substrate (grey) indicated, as well as the percentage of each species. Right panels show spectra from MALDI-TOF MS with peaks for acyl donor substrate (black) and product (orange) indicated, as well as the observed mass of each species.

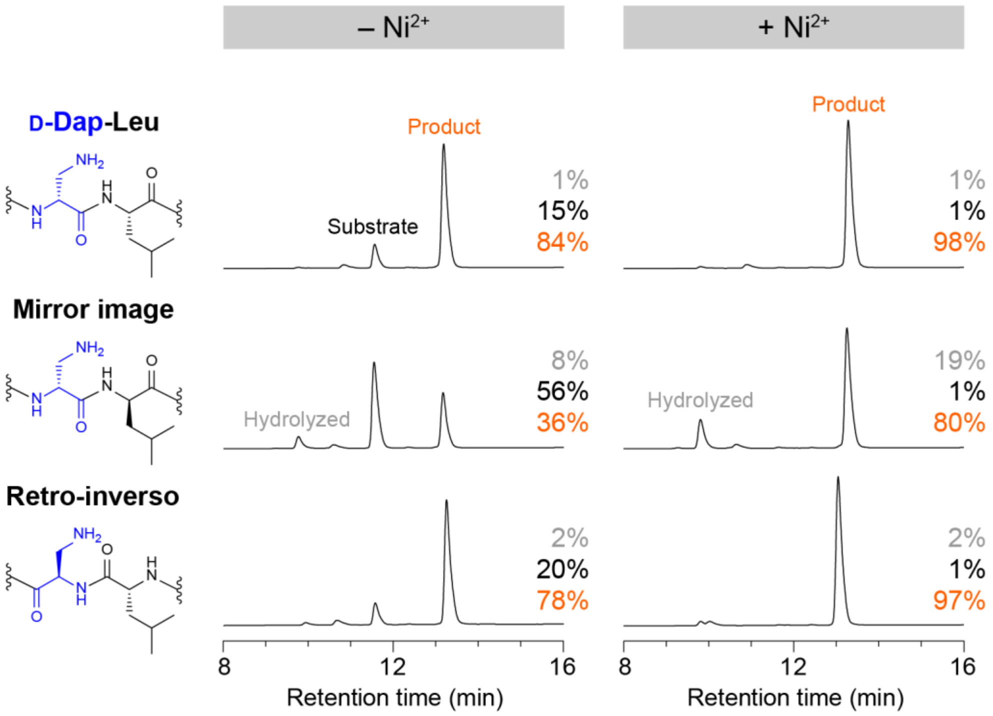

Figure S10. Isopeptide ligation with modified Dap substrates with or without GL byproduct quenching. Reactions were carried out using 500 µM Ac-G[D-Dap]LGV (D-Dap-Leu), Ac-G[D-Dap]lGv (mirror image) or Ac-vGl[D-Dap]G (retro-inverso), 100 µM acyl donor substrate (Ac-GWRNGLH) and 200 nM *Oa*AEP1 in 100 mM HEPES pH 7.5 (45 min at 25 °C). Additional reactions were run with 300 µM $NiCl_2$ (indicated by + Ni^{2+}) to quench the GLH byproduct released upon cleavage of the acyl donor substrate. Shown are analytical RP-HPLC traces (A280 nm). Peaks for acyl donor substrate (black), product (orange), and hydrolyzed substrate (grey) are indicated, as well as the percentage of each species. Spectra from MALDI-TOF MS are shown in Figure S11.

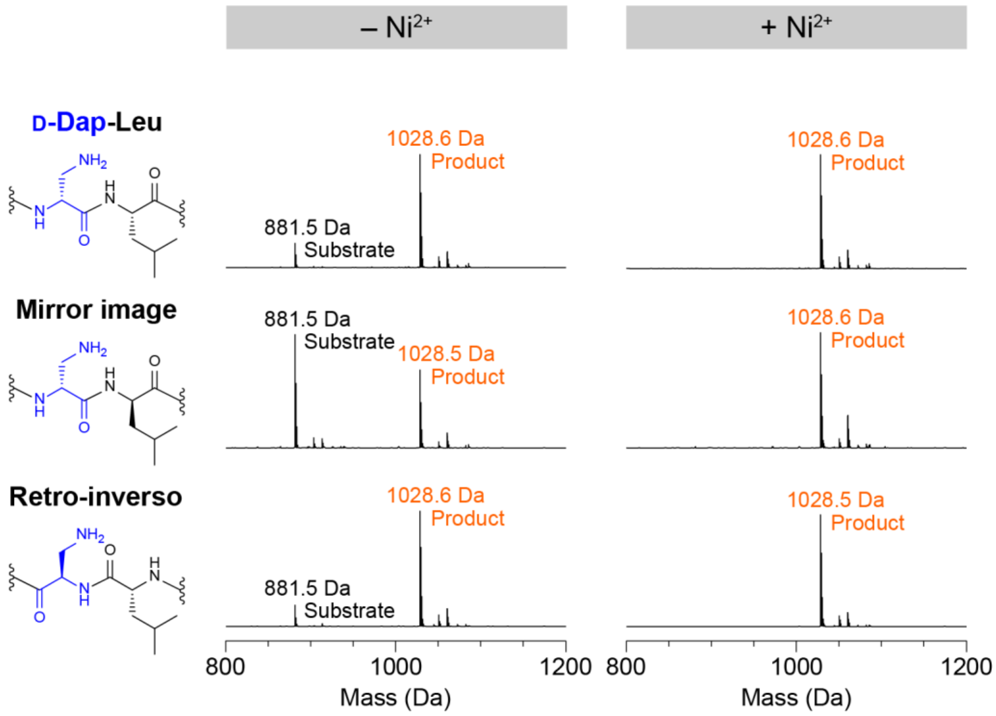

Figure S11. MALDI-TOF MS spectra for isopeptide ligation with modified Dap substrates with or without GL byproduct quenching. Reactions were carried out using 500 µM Ac-G[D-Dap]LGV (D-Dap-Leu), Ac-G[D-Dap]lGv (mirror image) or Ac-vGl[D-Dap]G (retro-inverso), 100 µM acyl donor substrate (Ac-GWRNGLH) and 200 nM *Oa*AEP1 in 100 mM HEPES pH 7.5 (45 min at 25 °C). Additional reactions were run with 300 µM $NiCl_2$ (indicated by + Ni^{2+}) to quench the GLH byproduct released upon cleavage of the acyl donor substrate. Peaks for acyl donor substrate (black) and product (orange) are indicated, as well as the observed mass of each species. Analytical RP-HPLC traces are shown in Figure S10.

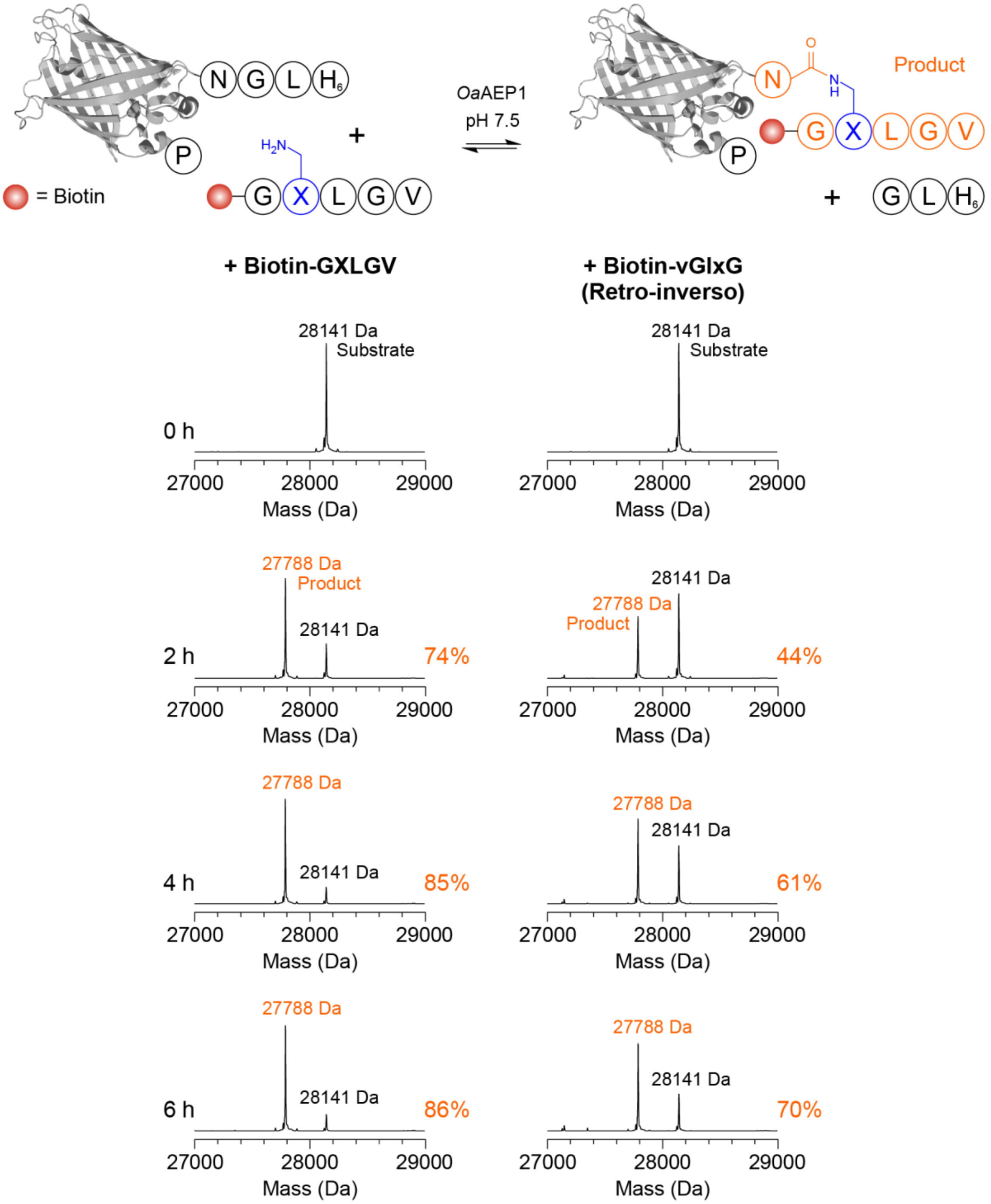

Figure S12. Additional time points for asparaginyl ligase-catalyzed isopeptide ligation at the C-terminus of a protein. eGFP with a C-terminal NGL-His$_6$ extension was labeled with biotin-G[Dap]LGV or biotin-vGl[D-Dap]G in reactions comprising 25 µM protein, 250 µM peptide (10 equiv.) and 500 nM *Oa*AEP1 in 100 mM HEPES pH 7.5 (reaction time as indicated). Shown are reconstructed spectra from ESI-MS with the observed substrate (black) and product (orange) masses indicated.

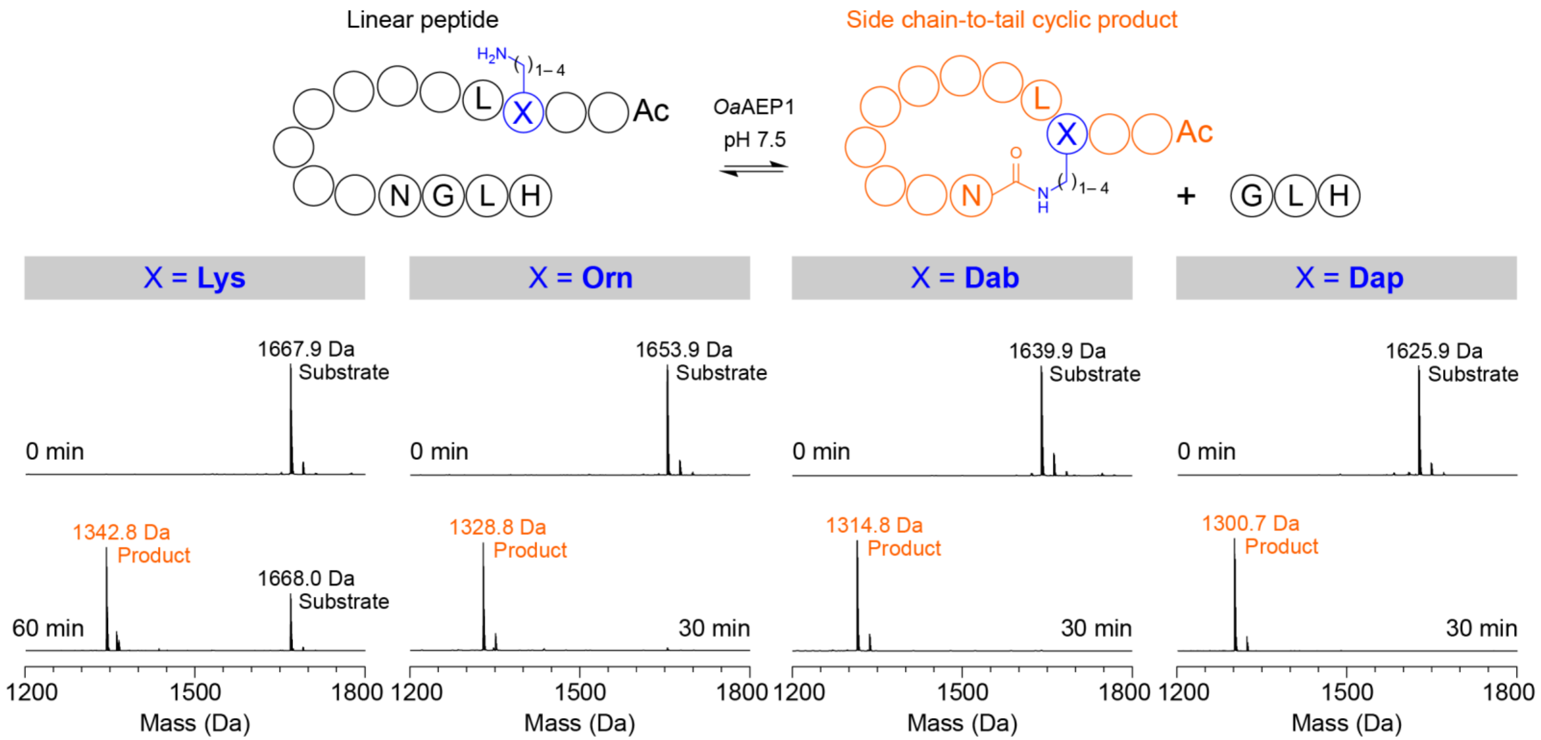

Figure S13. MALDI-TOF MS spectra for enzymatic production of isopeptide-linked macrocycles with cross-links of varying lengths. Peptides based on a linear template (sequence: Ac-YGXLTVpGLTRNGLH, p = D-Pro and X = Lys, Orn, Dab or Dap) were cyclized in reactions comprising 50 µM peptide and 100 nM *Oa*AEP1 in 100 mM HEPES pH 7.5 (30 or 60 min at 25 °C). Peaks for the linear peptide substrate and side chain-to-tail cyclic product are indicated, as well as the observed mass of each species. Analytical RP-HPLC traces are shown in Figure 2A.

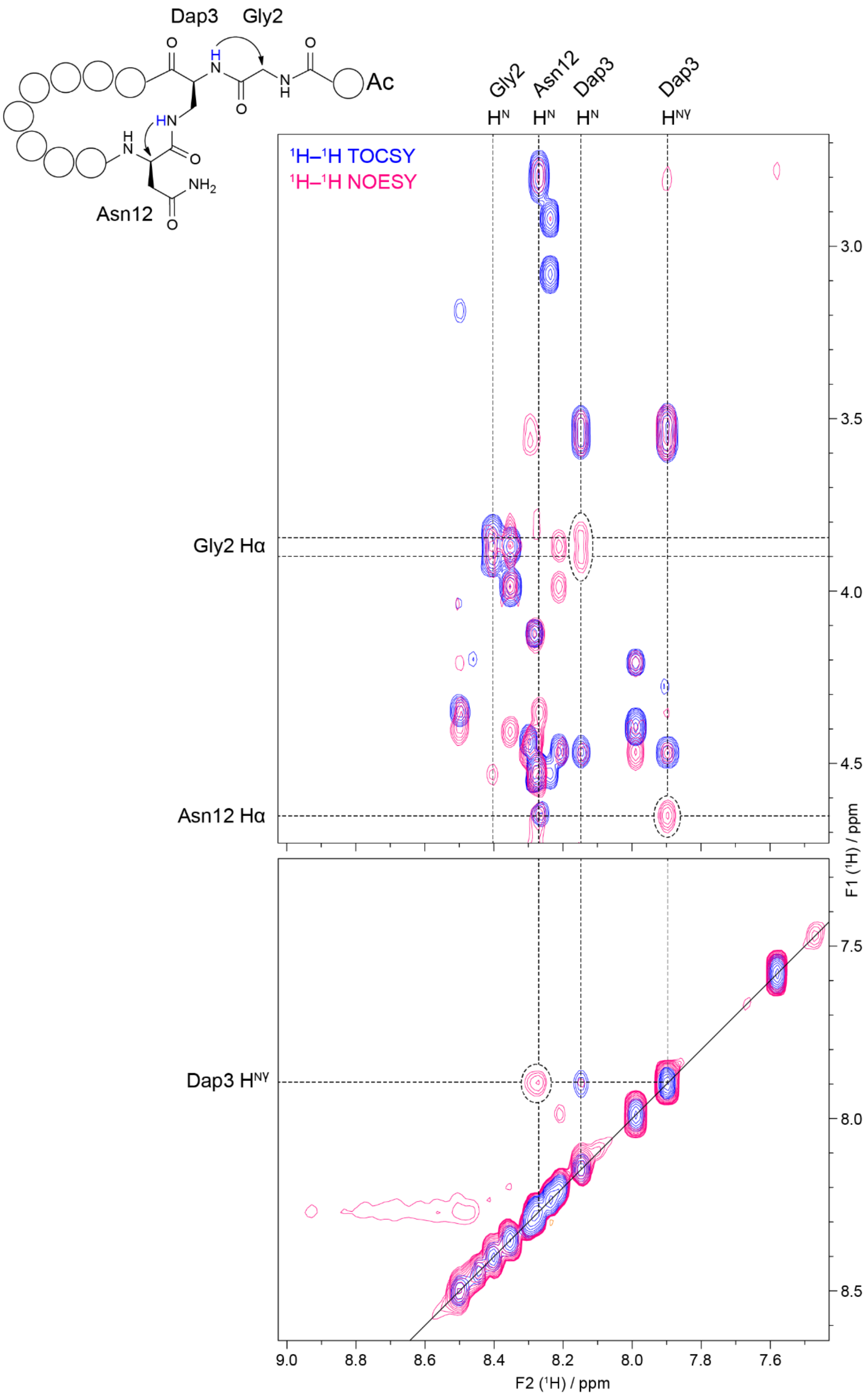

Figure S14. ^{1}H 2D NMR spectroscopy analysis of the side chain-to-tail cyclic product for the Dap β-hairpin peptide (Figure 2A, Figure S13). Shown are superimposed TOCSY (blue contours) and NOESY (pink contours) spectra of the amide region (7.5–9.0 ppm). Nuclear Overhauser effect cross-peaks are observed between the Dap3 Hγ proton and the Asn12 Hα and HN protons (circled), consistent with side chain-to-tail cyclization via an isopeptide bond. For the Dap3 NH proton, nuclear Overhauser effect cross-peaks are only observed with the Gly2 Hα2 and Hα3 protons (circled).

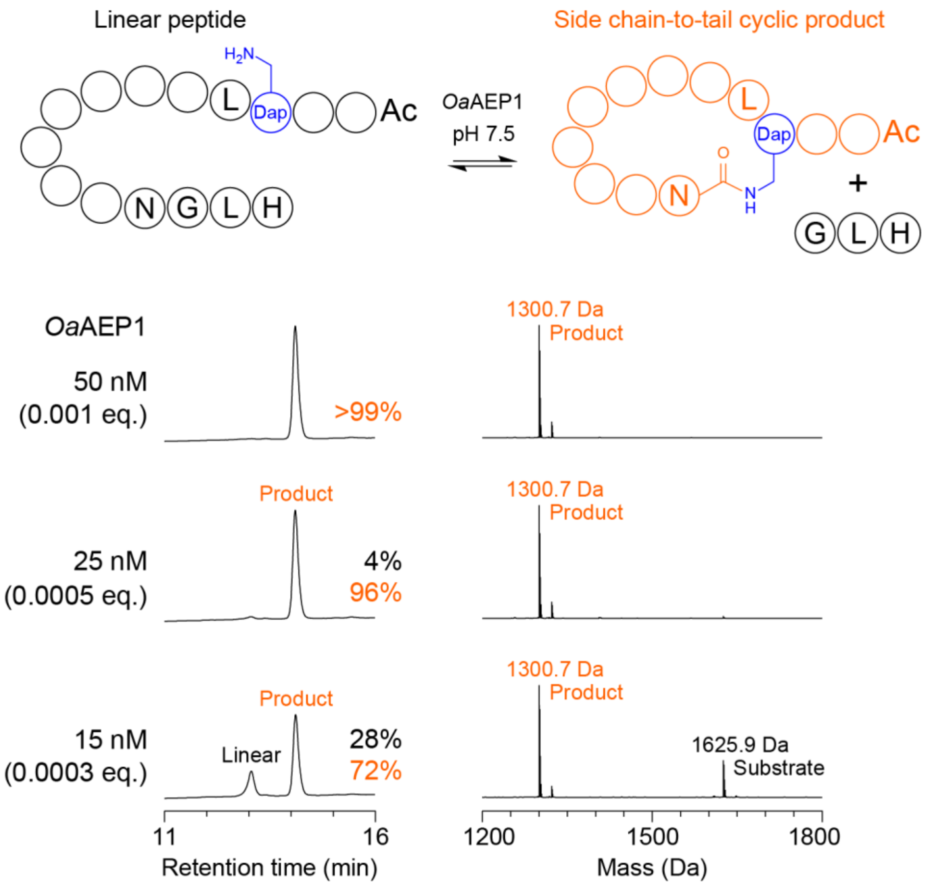

Figure S15. Enzymatic production of isopeptide-linked macrocycles using varying concentrations of *Oa*AEP1. A linear β-hairpin peptide (sequence: Ac-YG[Dap]LTVpGLTRNGLH, p = D-Pro) was cyclized in reactions comprising 50 µM peptide substrate and 15–50 nM *Oa*AEP1 (as indicated) in 100 mM HEPES pH 7.5 for 60 min at 25 °C. Left panels show analytical RP-HPLC traces (A280 nm) with peaks for linear peptide substrate (black) and product (orange) indicated, as well as the percentage of each species. Right panels show spectra from MALDI-TOF MS with peaks for linear peptide substrate (black) and product (orange) indicated, as well as the observed mass of each species.

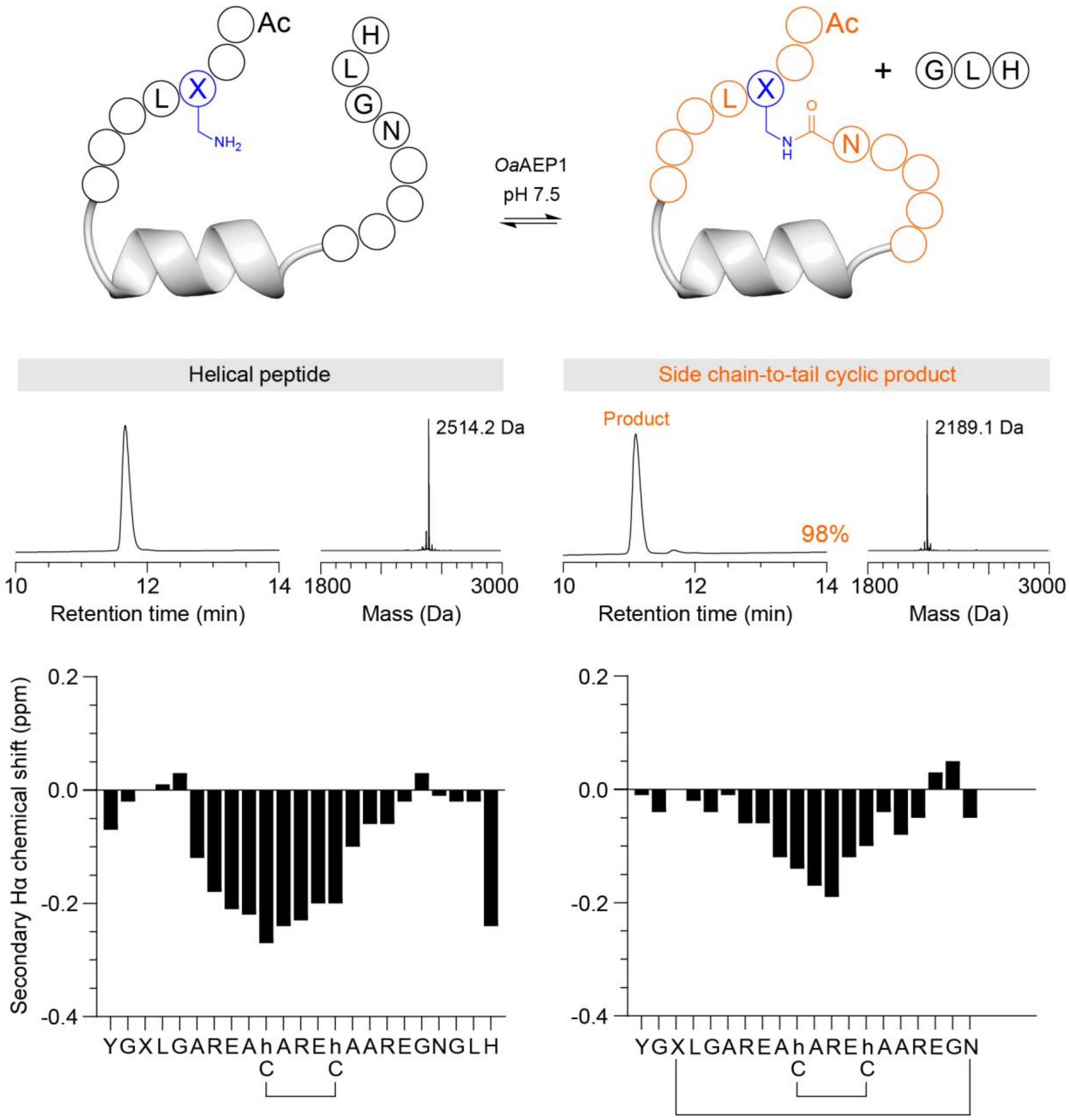

Figure S16. *Oa*AEP1-catalyzed side chain-to-tail cyclization of a stapled helical peptide. A dichloroacetone-stapled peptide with an N-terminal segment containing a Dap-Leu motif and a C-terminal NGLH recognition motif (sequence: Ac-YGXLGAREA[hCys]ARE[hCys]AAREGNGLH, X = Dap, peptide stapled at the hCys side chains) was cyclized via isopeptide ligation in reactions comprising 50 µM peptide and 200 nM *Oa*AEP1 in 100 mM HEPES pH 7.5 (60 min at 25 °C). Shown are analytical reverse-phase HPLC traces (A214 nm) and MALDI-TOF MS spectra of the purified stapled peptide substrate (left) and the crude reaction products from *Oa*AEP1-catalyzed isopeptide ligation to generate the side chain-to-tail cyclic product (right). The bottom panels show secondary Hα chemical shift measurements for the purified stapled peptide substrate (left) and side chain-to-tail cyclic product (right). Spectra were acquired on a Bruker Avance 600 MHz spectrometer equipped with a cryogenically cooled probe.

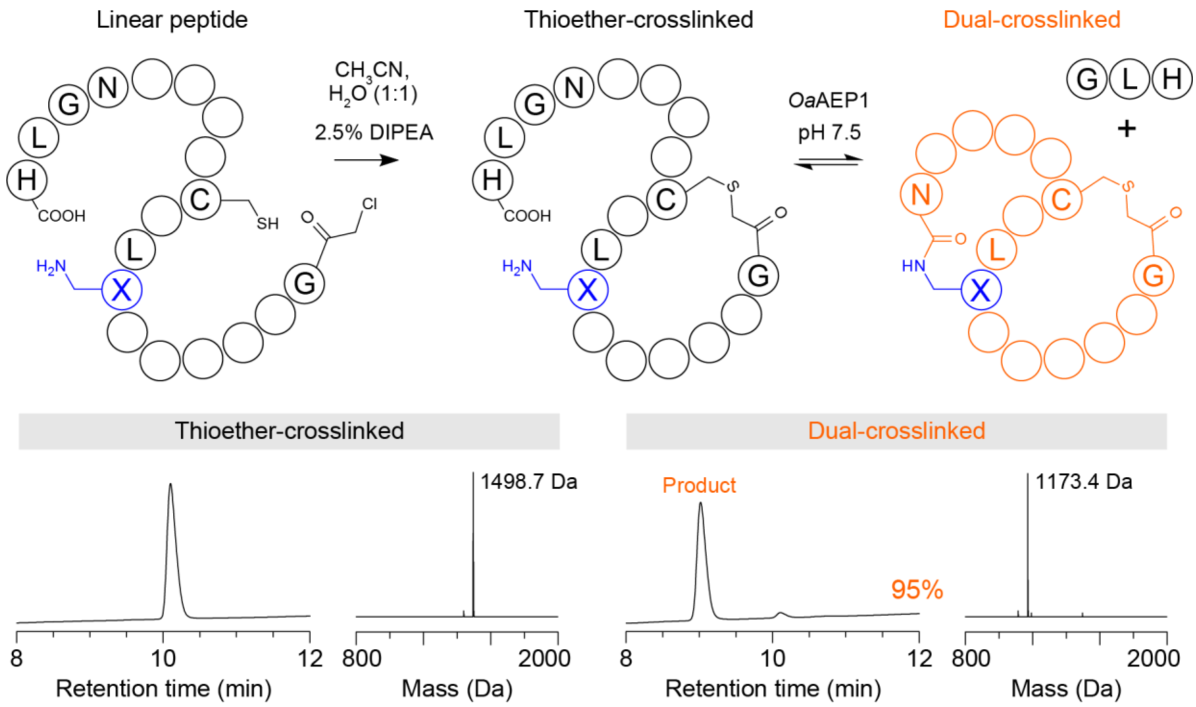

Figure S17. *Oa*AEP1-catalyzed formation of a bicyclic peptide topology. A linear peptide with an N-terminal segment containing a Dap-Leu motif and a C-terminal segment containing an NGLH extension was first head-to-side chain cyclized via a thioether cross-link between the terminal chloroacetyl-Gly residue and an internal Cys residue (sequence: *c*[GGASG[Dap]LGASGGC]GNGLH). The purified thioether cross-linked peptide served as a substrate for *Oa*AEP1-catalyzed isopeptide ligation in reactions comprising 50 µM peptide and 200 nM *Oa*AEP1 in 100 mM HEPES pH 7.5 (60 min at 25 °C) to form the dual cross-linked peptide. Shown are analytical reverse-phase HPLC traces (A214 nm) and reconstructed spectra from ESI-MS of the purified thioether cross-linked substrate (left) and the crude reaction products from *Oa*AEP1-catalyzed isopeptide ligation to generate the dual cross-linked product (right).

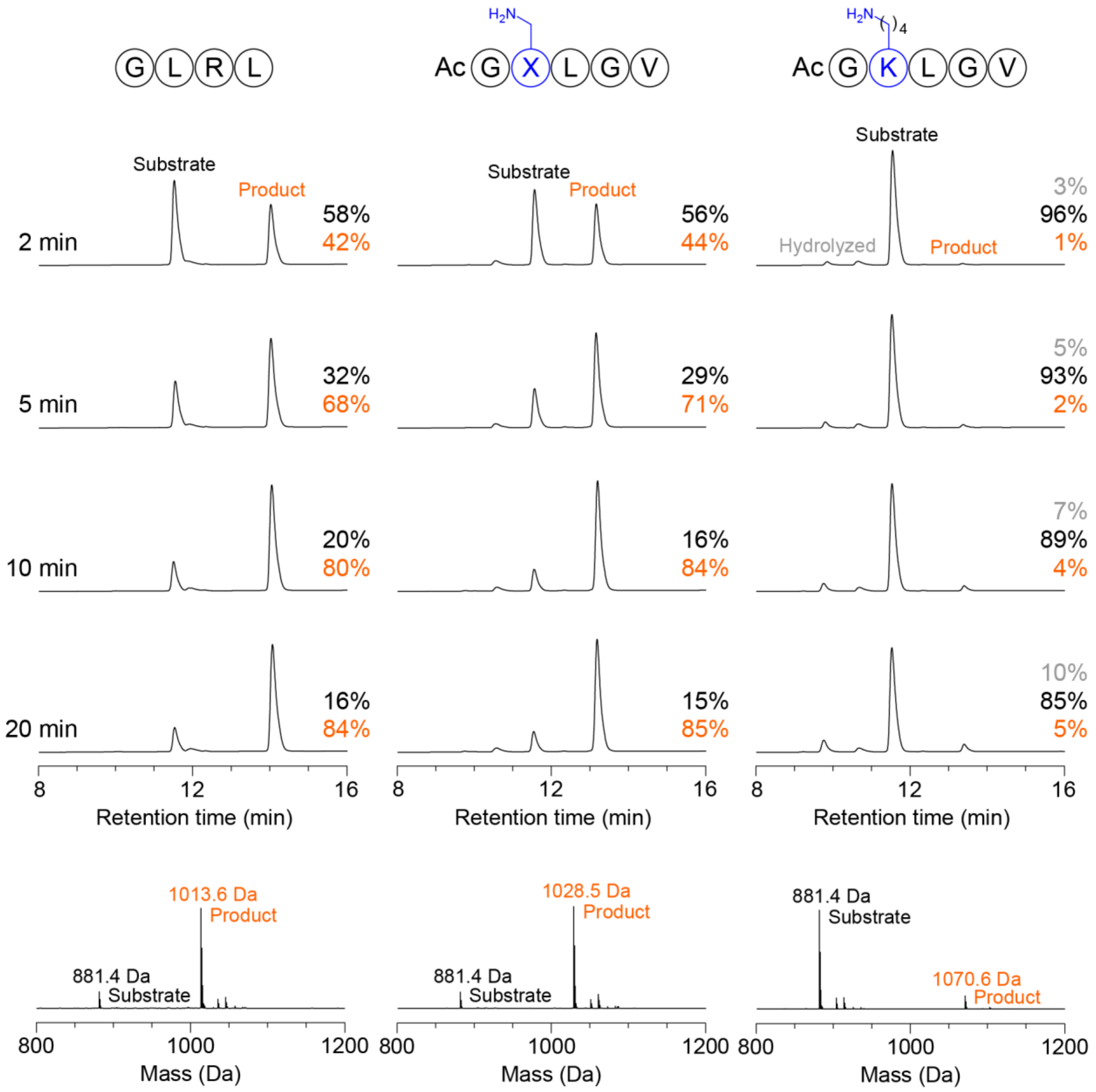

Figure S18. Time course for asparaginyl ligase-catalyzed isopeptide ligation at Dap-Leu compared to a conventional transpeptidation reaction at Gly-Leu. Reactions comprised 100 µM acyl donor substrate (Ac-GWRNGLH), 500 µM peptide nucleophile (GLRL, Ac-G[Dap]LGV or Ac-GKLGV, 5 equiv.) and 200 nM *Oa*AEP1 in 100 mM HEPES pH 7.5 (25 °C) and were quenched via TFA addition to 1% (v/v) after 2, 5, 10 or 20 min (indicated on the left). Shown are analytical RP-HPLC traces (A280 nm). Peaks for acyl donor substrate (black), product (orange), and hydrolyzed substrate (grey) are indicated, as well as the percentage of each species. The bottom panels show spectra from MALDI-TOF MS for the reaction endpoint (20 min). Peaks for acyl donor substrate (black) and product (orange) are indicated, as well as the observed mass of each species.

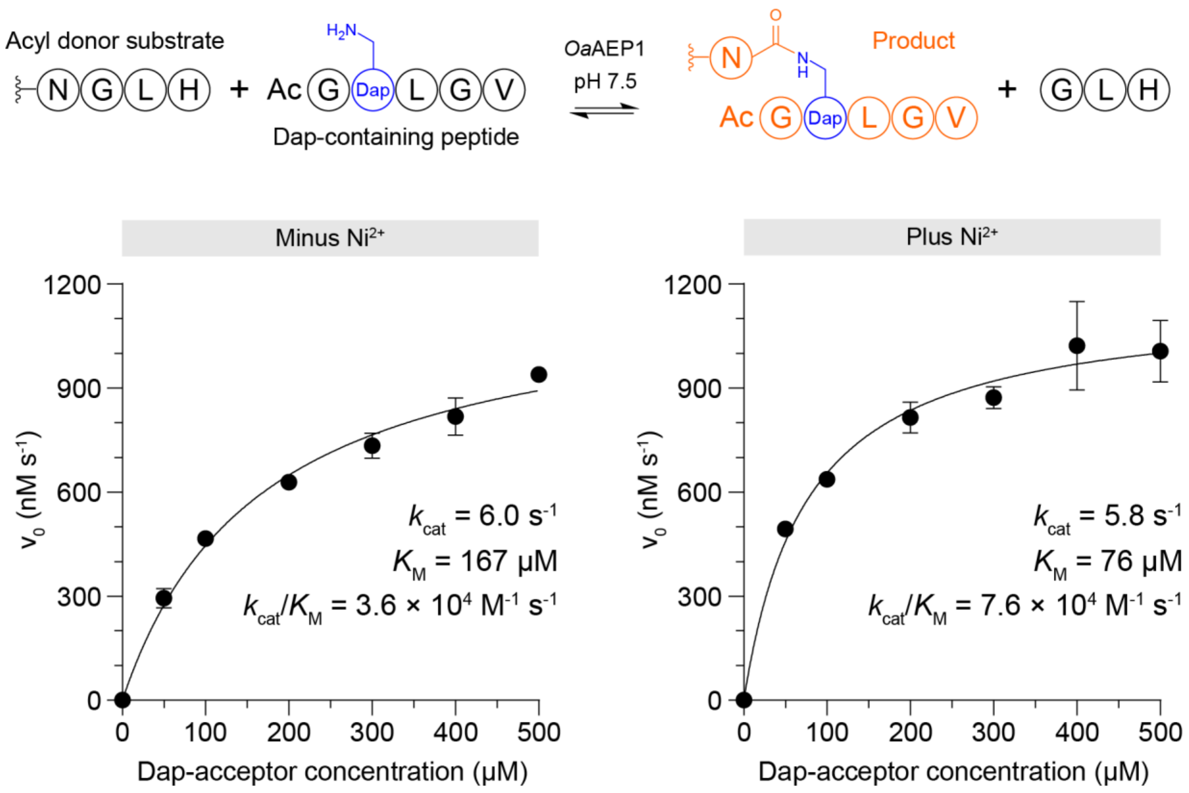

Figure S19. Michaelis-Menten kinetics for *Oa*AEP1-catalyzed isopeptide ligation at Dap. Initial velocities (v_0) were determined, via RP-HPLC analysis of product formation, for reactions comprising 1 mM NGLH-containing acyl donor substrate (Ac-GWRNGLH), 0-500 µM Dap-containing acceptor peptide (Ac-G[Dap]LGV) and 200 nM *Oa*AEP1 in 100 mM HEPES buffer pH 7.5 (25 °C), with or without 2 mM $NiCl_2$ to quench the released GLH byproduct (as indicated above each graph). Data points represent the mean from three independent experiments and errors bars indicate the standard deviation. Best-fit values for k_{cat} and K_M were determined by fitting the Michaelis-Menten model in GraphPad Prism 10.

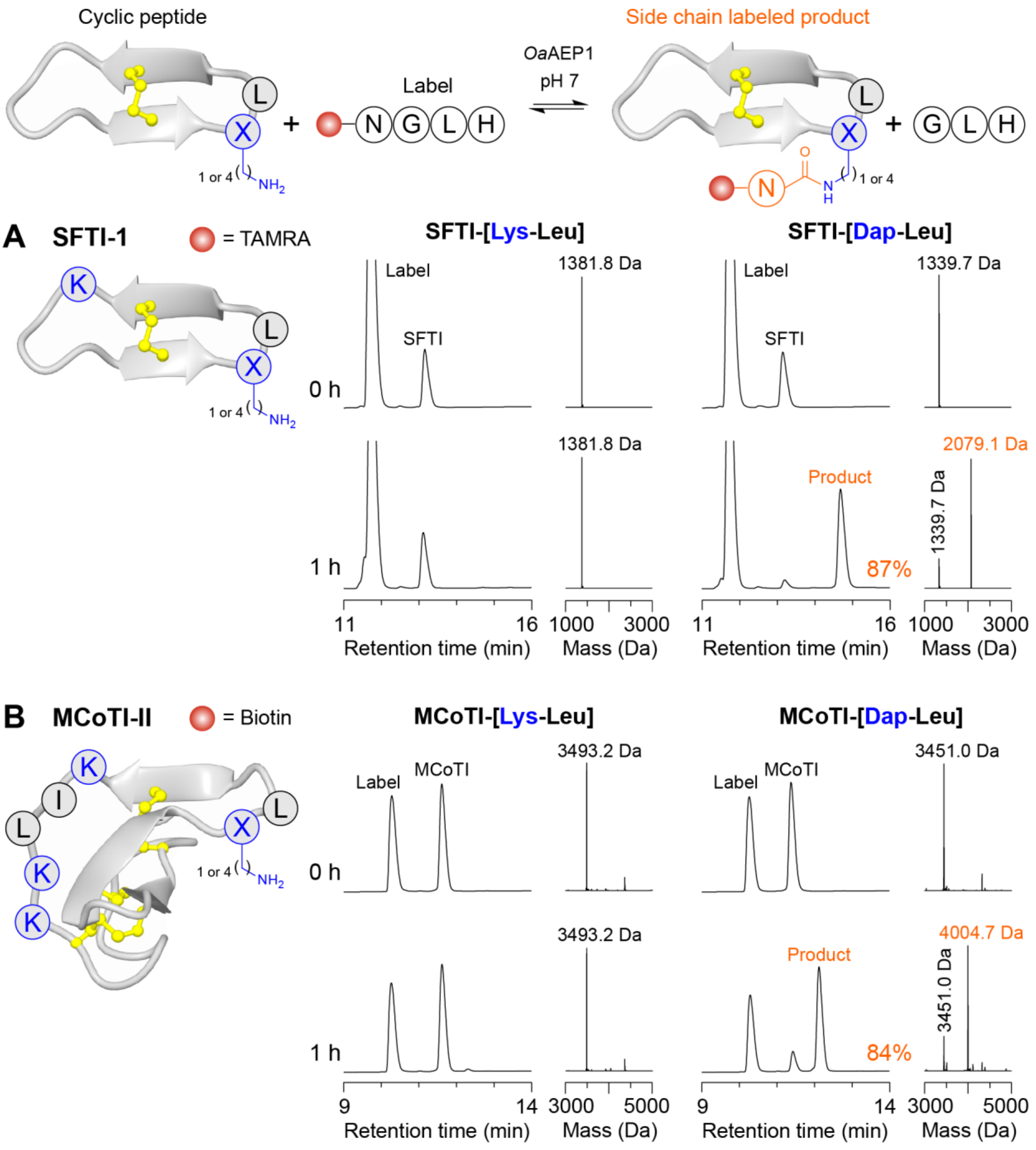

Figure S20. Efficient, site-selective labeling of Lys-containing cyclic peptides via isopeptide ligation at Dap. Labeling of (A) the 14-amino acid cyclic peptide SFTI-1 containing a Lys-Leu or Dap-Leu motif using a TAMRA-GRNGLH peptide, and (B) the 34-amino acid cyclic knottin MCoTI-II containing a Lys-Leu or Dap-Leu motif using a biotin-GRNGLH peptide. Reactions comprised 100 µM cyclic peptide, 400 µM label (4 equiv.) and 200 nM OaAEP1 in 100 mM HEPES pH 7 (60 min at 25 °C). Shown are analytical RP-HPLC traces (A214 nm) before addition of enzyme and after 1 h incubation. Peaks for unlabeled cyclic peptide (SFTI or MCoTI), label, and product are indicated. Conversion to product for Dap-Leu analogs was calculated by measuring consumption of the unlabeled cyclic peptide via peak integration at t = 0 h and 1 h. Reconstructed spectra from ESI-MS of the crude reaction products are also shown, with the observed masses for unlabeled cyclic peptide (black) and product (orange) indicated.

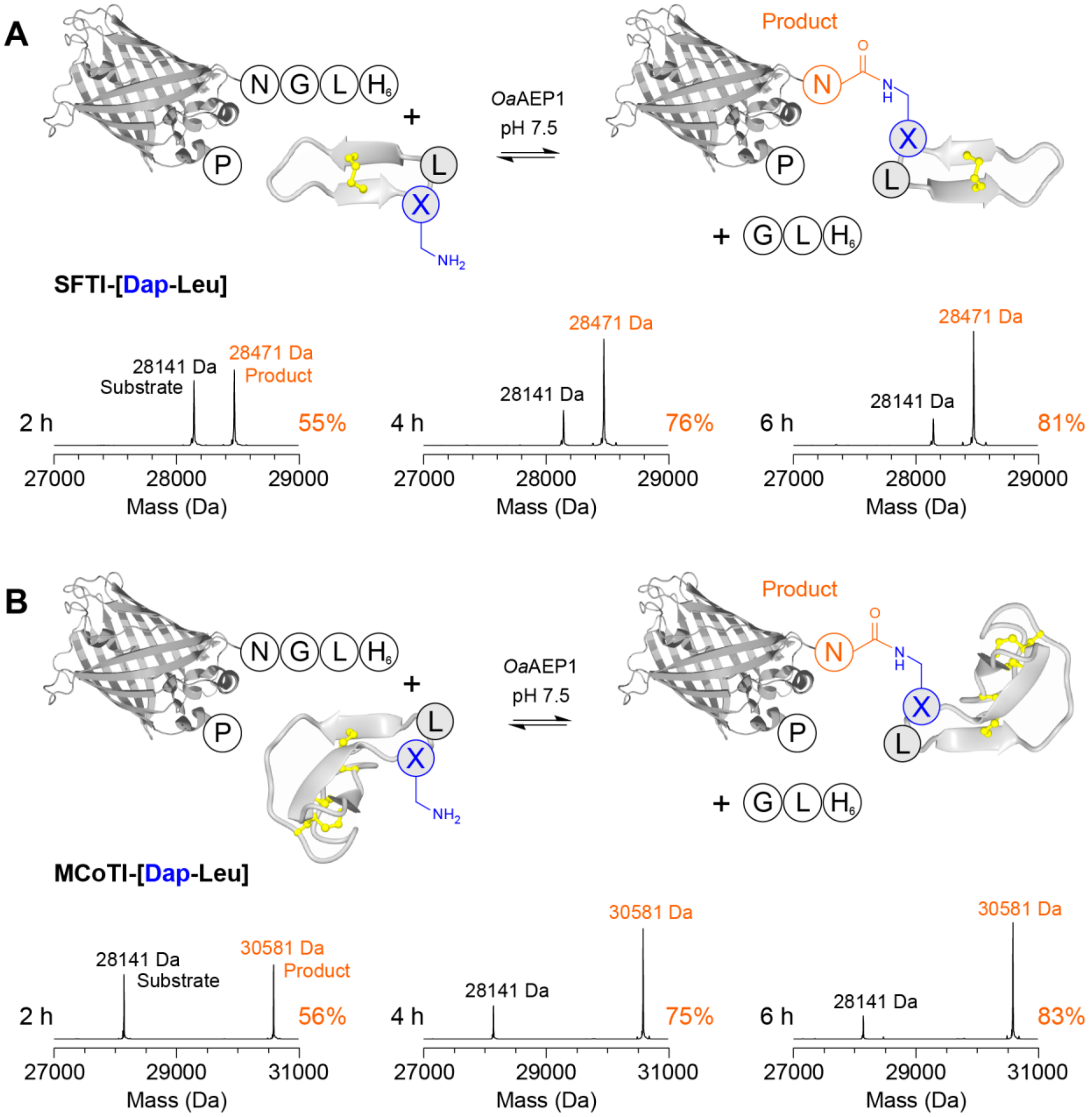

Figure S21. Additional time points for protein-cyclic peptide fusion via *Oa*AEP1-catalyzed isopeptide ligation. Direct conjugation of (A) the cyclic peptide SFTI-1 containing a Dap-Leu motif or (B) the cyclic knottin MCoTI-II containing a Dap-Leu motif, to eGFP containing a C-terminal NGL-His$_6$ extension. Reactions comprised 25 µM protein, 250 µM cyclic peptide (10 equiv.) and 500 nM OaAEP1 in 100 mM HEPES pH 7.5 (reaction time as indicated). Shown are reconstructed spectra from ESI-MS with the observed substrate (black) and product (orange) masses indicated.
